# Toward reliable thalamic segmentation: an evaluation of automated methods for structural MRI

**DOI:** 10.1007/s00429-026-03163-z

**Published:** 2026-07-18

**Authors:** Georgios P. D. Argyropoulos, Christopher R. Butler, Manojkumar Saranathan

**Affiliations:** 1 Memory Research Group, Nuffield Department of Clinical Neurosciences, University of Oxford, Oxford, UK; 2 Division of Psychology, Faculty of Natural Sciences, University of Stirling, Stirling, FK9 4LA UK; 3 Department of Brain Sciences, Imperial College London, London, W12 0NN UK; 4 Departamento de Neurología, Pontificia Universidad Católica de Chile, Avda. Libertador Bernando O’Higgins 340, Santiago, Chile; 5 Department of Radiology, University of Massachusetts Chan Medical School, Worcester, MA USA

**Keywords:** Thalamus, Segmentation, THOMAS, HIPS-THOMAS, FreeSurfer, MRI

## Abstract

Automated thalamic nuclear segmentation has contributed towards a shift in neuroimaging analyses, from treating the thalamus as a homogeneous, passive relay, to a set of individual nuclei, embedded within distinct brain-wide circuits. However, many studies continue to widely rely on FreeSurfer’s segmentation of T1-weighted structural MRIs, despite their poor intrathalamic nuclear contrast. Meanwhile, a convolutional neural network tool has been developed for FreeSurfer, using information from both diffusion and T1-weighted MRIs. Another popular thalamic nuclear segmentation technique is HIPS-THOMAS, a multi-atlas-based method that leverages white-matter-like contrast synthesized from T1-weighted MRIs. However, comparisons amongst methods remain scant, and the thalamic atlases against which these methods have been assessed have their own limitations. These issues may compromise the quality of cross-species comparisons, structural and functional connectivity studies in health and disease, as well as the efficacy of neuromodulatory interventions targeting the thalamus. Here, we report, for the first time, comparisons amongst HIPS-THOMAS, the standard FreeSurfer segmentation, and its more recent development, against two thalamic atlases. We used two cohorts of healthy adults, and one cohort of patients in the chronic phase of autoimmune limbic encephalitis. In healthy adults, HIPS-THOMAS surpassed, not only the standard FreeSurfer segmentation, but also its more recent, diffusion-based update. The improvements made with the latter were limited to a few nuclei. Finally, the standard FreeSurfer method underperformed in distinguishing between patients and healthy controls based on the affected anteroventral and pulvinar nuclei. We provide recommendations on automated segmentation methods of the human thalamus using structural brain imaging.

**Supplementary Information:**

The online version contains supplementary material available at https://doi.org/10.1007/s00429-026-03163-z.

## Introduction

There is a growing need for neuroimaging studies to stop treating the thalamus as a single, homogeneous unit, and instead examine its nuclei as separate, highly specialised structures supporting multiple cognitive processes (Segobin et al. 2024). Until recently, distinguishing human thalamic nuclei was only feasible via post-mortem histology or manual delineation of ex vivo MRIs. While the segmentation of all nuclei (*n* > 60) remains beyond the capacity afforded by current MRI resolution, developments since the second half of the last decade have enabled the automated segmentation of several nuclei from diffusion, functional, and structural MRI data (Battistella et al. 2017; van Oort et al. 2018; Iglesias et al. 2018; Su et al. 2019; Umapathy et al. 2022; Tregidgo et al. 2023; Vidal et al. 2024).

The most widespread of these methods is the Bayesian-based segmentation of T1w-MRI incorporated into *FreeSurfer* tool (FS-T1), which is based on probabilistic maps of thalamic nuclei determined from ex vivo MRIs and post-mortem histology samples (Iglesias et al. 2018). It has been used, not only for volumetry (Shin et al. 2019; Lee et al. 2020; Xiong et al. 2021; Trufanov et al. 2021; Nakhid et al. 2023; Mørch-Johnsen et al. 2023), but also for ROI definition in task-based fMRI analyses (Gentile et al. 2024), and functional (Velioglu et al. 2023) and structural connectivity analyses (Liu et al. 2022). Another popular method, especially in the neurosurgical world, is THalamus Optimized Multi-Atlas Segmentation method (Su et al. 2019) or THOMAS. This multi-atlas method leverages the improved intrathalamic contrast of white-matter nulled (WMn) T1-w MRI sequences (Su et al. 2019) like Fast Grey ATtenuated Inversion Recovery (FGATIR). A recent variant uses HIstogram-based Polynomial Synthesis (HIPS), which synthesizes WMn image contrast from standard T1w-MRIs. This method, called “HIPS-THOMAS” (Vidal et al. 2024), eliminates the need for specialized sequences, enabling analysis of data from existing public databases. WMn-MRI has been shown to generate more accurate segmentation (Umapathy et al. 2022) relative to T1w-MRIs.

A more recent development has been the convolutional neural network tool for FreeSurfer (Tregidgo et al. 2023) (FS-DTI), which uses information from both diffusion and T1w-MRI (we refer to this as “FS-DTI”). Given its implementation in FreeSurfer and the improved visualization of ventral and medial thalamic boundaries, we expect this method to be more accurate and more readily adopted by the community (Soskic et al. 2025; Argyropoulos et al. 2026; Wang et al. 2026; Baird et al. 2026).

Despite these advances, there is no consensus on the thalamic nuclei that can be reliably segmented or the techniques that should be leveraged to enhance their delineation (Segobin et al. 2024). While validation analyses of FS-T1 have showed poor results (Su et al. 2019; Williams et al. 2022) and lower quality relative to HIPS-THOMAS for several nuclei (Williams et al. 2024), comparisons between approaches are scant (Iglehart et al. 2020; Williams et al. 2024). Importantly, the two state-of-the-art structural-based methods- HIPS-THOMAS and FS-T1- have not been compared with the recent FS-DTI improvement. Moreover, the multiplicity of methods available and the lack of objective performance measures leave room for cherry-picking (Segobin et al. 2024). Comparative neuroanatomical studies, robust replication of findings in cognitive/clinical neuroscience, as well as favourable treatment outcomes of neuromodulatory interventions targeting the thalamus are all dependent on the quality of thalamic segmentation methods.

Gold-standard manual segmentations (e.g., Su et al. 2019) are not easily attainable when comparing automated segmentation methods on large datasets. For this reason, the Krauth-Morel atlas (henceforth “Morel”; Krauth et al. 2010), a digital representation of the Morel stereotactic atlas (Morel et al. 1997; Morel 2007) brought to MNI152 space (Jakab et al. 2012), is often used as a “silver-standard” reference by many authors (Williams et al. 2022, 2024). One disadvantage of the Morel atlas is the limited series of post-mortem data (9 thalamic blocks from 5 normal human brains) used in its construction, representing little variability in anatomy. More relevantly, recent work has highlighted limitations in the quality of Morel (the MNI-space MRI atlas as opposed to the original histological), especially for segmentations interfacing with large white matter bundles or cerebrospinal fluid (Brun et al. 2022). Brun and colleagues (Brun et al. 2022) also proposed a new “7TAMIbrainDGN” atlas (henceforth, “Marseille atlas”), which contains 24 deep grey matter (including 12 thalamic) nuclei per hemisphere on their high-contrast/resolution T1w 7TAMIbrainT1w_30 template.

In this work, we conducted rigorous performance comparisons of two Freesurfer-based thalamic segmentation variants- FS-T1 and FS-DTI-, and HIPS-THOMAS, in two separate cohorts of healthy subjects. We evaluated them against two thalamic atlases: Morel and Marseille. Lastly, we also compared FS-T1, FS-DTI, and HIPS-THOMAS on their efficacy in distinguishing between healthy adults and patients in the chronic phase of autoimmune limbic encephalitis - a non-neurogenerative condition that is mainly associated with inflammation and subsequent atrophy in medial temporal lobe structures, and has been shown to impact the structural integrity of the thalamus (Qiao et al. 2025; Wagner et al. 2015; Argyropoulos et al. 2019, 2026; Loane et al. 2019). This comparison eliminates the use of atlases and the concomitant errors from registration and directly focuses on the ability of the methods to accurately and sensitively discriminate healthy individuals from those with disease.

## Materials and methods

### Datasets

#### Healthy controls: Memory and Amnesia Project (MAP)

The first cohort comprised 35 healthy adults (age: mean = 55.14; SD = 14.04 years; 25 M:10 F), recruited in the Memory and Amnesia Project (Nuffield Department of Clinical Neurosciences, University of Oxford) (Argyropoulos et al. 2026), referred to as “MAP35”. They were all fluent speakers of English, with no known history of neurological or psychiatric issues, no abnormalities detected in their structural MRIs, and no deficits detected in their neuropsychological assessment (Argyropoulos et al. 2019, 2020).

T1w-MRIs were acquired with a Siemens 3T Trio system and a 32-channel head coil (University of Oxford Centre for Clinical Magnetic Resonance Research), using a MPRAGE sequence (echo time = 4.7 ms, repetition time = 2040 ms, 8° flip angle, field of view = 192 × 192 mm, voxel size = 1 × 1 × 1 mm). Diffusion MRI involved a single-shot EPI sequence (64 slices; slice thickness = 2 mm, 0 gap; axially acquired; 64 directions at b = 1,500 s/mm^2^, repetition time = 8,900 ms; echo time = 94.8 ms; voxel size = 2 × 2 × 2 mm; field of view = 192 × 192 mm), along with a no-diffusion-weighted image (b = 0 s/mm^2^).

#### Healthy controls: Human Connectome Project (HCP)

To replicate our findings with a publicly available MRI dataset of equivalent size, the “MGH HCP Adult Diffusion” dataset of the Human Connectome Project was selected. This dataset comprises diffusion and structural imaging data from 35 healthy young adults with no known history of major psychiatric or neurological disorders (age: range = 20–59; estimated mean = 31.57; estimated SD = 8.52; 16 F:19 M), referred to as “HCP35”.

T1w-MRIs were acquired with a customized Siemens 3T Connectom scanner (modified 3T Skyra system) and a 64-channel, tight-fitting brain array coil, in the MGH/HST Athinoula A. Martinos Center for Biomedical Imaging. A MPRAGE sequence was used (echo time = 1.15 ms, repetition time = 2,530 ms, 7° flip angle, field of view = 256 × 256 mm, voxel size = 1 × 1 × 1 mm). Diffusion MRI involved a spin-echo EPI sequence (96 slices; slice thickness = 1.5 mm; axially acquired; 5 runs, with b = 1,000–10,000 s/mm^2^, and 64–128 directions; repetition time = 8,800 ms; echo time = 57 ms; voxel size = 1.5 × 1.5 × 1.5 mm; field of view = 210 × 210 mm), along with one non-diffusion weighted (b = 0) image being collected every 14 image volumes. Details on T1w-MRI and Diffusion MRI scans and preprocessing can be found here: https://humanconnectome.org/study/hcp-young-adult/document/mgh-adult-diffusion-data-acquisition-details/.

Of those, however, 2 subjects were excluded (*mgh_1035*, as this dataset lacked preprocessed data, and *mgh_1020*, as the FS-DTI segmentation failed upon inspection, also reflected in the total thalamic volumes estimated by each method - Supplementary Table 1).

### Patient data

Finally, we used data from a cohort of 38 patients in the chronic phase of autoimmune limbic encephalitis, which is primarily characterised by hippocampal atrophy and residual episodic memory impairment (Argyropoulos et al. 2019). One patient’s diffusion MRI data were not acquired, hence 37 patients’ data were analysed (26 M:11 F; age at imaging: mean = 61.14; SD = 14.01 years; controls vs. patients: M:F ratio: χ^2^ = 0.01, *p* = 0.914; age at imaging: t(70) = 1.81, *p* = 0.074). This is a well characterised cohort, whose (acute) clinical and (postacute) neuropsychological details have been presented previously (Argyropoulos et al. 2019, 2020). They were all assessed by a single neurologist (CRB) prior to inclusion in the study. They had all undergone MRI at the time of initial clinical presentation along with neuropsychological assessment at the Russell Cairns Unit, Oxford, UK (2013–2018). They had been diagnosed with this condition according to established diagnostic criteria (Graus et al. 2016), and they had no history of previous psychiatric or neurologic disorder that could have resulted in cognitive impairment. None of them had presented with positive PCR testing for herpes simplex virus or with anti-NMDAR encephalitis. The patients were also scanned using the protocol of the MAP35 dataset (Argyropoulos et al. 2026).

### Native-space segmentation

#### Freesurfer-T1

The output of FreeSurfer’s (v. 7.4.1-20230614-7eb8460) “*recon-all*” was employed (T1w-MRI parcellation; command: “*recon-all -i T1.nii.gz -s T1 -all*”) to segment the thalamic nuclei with the algorithm (“*segmentThalamicNuclei.sh*”; command: “*segment_subregions thalamus --cross T1*”) described in Iglesias et al. (2018). Thalamic segmentations were converted from FS to native space (“*mri_label2vol*”), and from “.mgz” to “.nii” format (“*mri_convert*”).

#### Freesurfer-DTI

For FS-DTI (Tregidgo et al. 2023), the following inputs were provided to the command (“*mri_segment_thalamic_nuclei_dti_cnn --t1 norm.mgz --aseg aseg.mgz --fa dtifit_FA.nii.gz --v1 dtifit_V1.nii.gz --o thal_cnn_outputSegmentation.nii.gz --vol thal_cnn_volumes.csv --model /usr/local/freesurfer/7.4.1/models/thalseg_1.1.h5 --threads 3*”): a bias-corrected, whole-brain structural T1w-MRI (“norm.mgz”), a whole-brain segmentation (“aseg.mgz”- output from “*recon-all*”), a fractional anisotropy volume (“dtifit_FA.nii.gz”) and a 4D volume containing the principal direction vector for each DTI voxel (“dtifit_V1.nii.gz”). The last two were derived with TRACULA (TRActs Constrained by UnderLying Anatomy (Yendiki et al. 2011; Maffei et al. 2021) for MAP35, and with DTIFit in FSL (v. 6.0.7.8) for HCP35, as the data from the latter dataset were already preprocessed with gradient non-linearity correction, motion correction, eddy-current correction, b-vector correction (https://humanconnectome.org/study/hcp-young-adult/document/mgh-adult-diffusion-data-acquisition-details/; note that TRACULA also calls DTIFit: https://surfer.nmr.mgh.harvard.edu/fswiki/FsTutorial/TraculaOutputs). The algorithm also used the FS-T1-based segmentation. Each subject’s norm.mgz was registered, without resampling, to diffusion space.

#### HIPS-THOMAS

HIPS-THOMAS v.2 (command: “*docker run -it --rm --name sthomas -v ${PWD}:/data -w /data --user ${UID}:$(id -g) anagrammarian/sthomastest hipsthomas.sh -v -t1 -i T1.nii.gz*”) was used to segment the thalamus.

The segmented nuclei that comprise the output of each method are detailed in Supplementary Methods 3.

### Segmentation preparation

To enable direct comparisons amongst the three methods, FS segmentations and Morel ROIs were harmonized to match the nomenclature employed in HIPS-THOMAS, following the methodology described earlier in (Williams et al. 2024). Note that both Freesurfer and HIPS-THOMAS nuclei follow the Morel nomenclature and the harmonization did not change fundamental ontology or boundary definitions but merely combined subdivisions like those of pulvinar and mediodorsal which are present in Freesurfer variants but not in HIPS-THOMAS. Segmentations not generated by HIPS-THOMAS (e.g., laterodorsal) or FS-T1/FS-DTI (e.g., habenula) were omitted. This resulted in 22 segmentations (11/hemisphere) (Supplementary Table 2). These comparisons were repeated using the Marseille atlas as a second reference. Note that the Marseilles atlas also uses Morel as a reference to parcellate the thalamic nuclei following the same nomenclature and boundaries, making it consistent. However, this atlas is driven more directly by MRI contrast than histological information, making it more appealing for MRI-based segmentation. As the Marseille atlas was in its own reference space, the thalamic nuclei of interest from this atlas were warped, as above, to FSL’s MNI152_T1_1mm_brain for the purposes of comparing the three segmentation methods (Supplementary Table 3).

Following thalamic segmentation in native space, each T1w-MRI underwent brain extraction (FSL’s BET), and an affine matrix was calculated with FSL’s FLIRT (Jenkinson et al. 2002) for rigid/affine linear registration to MNI space (initial global alignment; template: MNI152_T1_1mm_brain: https://git.fmrib.ox.ac.uk/fsl/data_standard/-/raw/master/MNI152_T1_1mm_brain.nii.gz?ref_type=heads&inline=false ). Using that affine matrix, nonlinear registration was conducted for the T1w-MRI to MNI space (FSL’s FNIRT; Andersson et al. 2008), generating warps from native to MNI space and from MNI to native space. Using nearest-neighbour interpolation, the former was applied to register native-space segmentations from all three methods to MNI space, and the latter was used for all Morel ROIs in MNI space (Krauth et al. 2010; Jakab et al. 2012), separately for each subject of each dataset, bringing them to each subject’s native anatomical space. The same warps were used when comparisons were repeated with the Marseille atlas.

### Statistical analysis

Analysis was performed in both native-space and MNI-space. We used two complementary metrics: Dice coefficients and Average Hausdorff Distances (AHDs). The Dice coefficient, an overlap-based metric, is defined as *“Dice(S*, R) = 2*[(‖S∩R‖)/(‖S‖+‖R‖)]”, wherein *“S”* is a certain segmentation from FS-T1, FS-DTI, or HIPS-THOMAS, *“R”* is the corresponding reference Morel/Marseille segmentation in the same space, *“S∩R”* corresponds to the set of voxels that are shared by S and R, and *“‖‖”* measures the number of voxels in the segmentation. As elsewhere (Pajula et al. 2012; Williams et al. 2024), the following value cut-offs were used to discuss segmentation accuracy: Dice = 0: “no agreement”; 0 < Dice < 0.2: “slight agreement”; 0.2 ≤ Dice < 0.4: “fair agreement”; 0.4 ≤ Dice < 0.6: “moderate agreement”; 0.6 ≤ Dice < 0.8: “substantial agreement”; 0.8 ≤ Dice ≤ 1: “almost perfect agreement”. The Hausdorff-distance metric helps address the issue of overlap-based metrics disproportionately penalising smaller structures (Taha and Hanbury 2015; Williams et al. 2024). Here it was defined it as *“AHD(S*,* R) = max(d(S*,* R)*,*d(R*,* S)”*, where “*d*(*S*,*R*)” is the average minimum distance (min ‖*s* − *r*‖) from voxels in S to R, “*d*(*R*, *S*)” is the average minimum distance (min ‖*r* − *s*‖) from voxels in R to S, and “*max*” refers to the largest of these two values (d(R, S), d(S, R) being selected. Similar to Williams et al. (2024), segmentations with AHD≤1 mm are treated as being of sufficiently small distance from the corresponding reference Morel/Marseille segmentation.

A series of one-way repeated-measures ANOVAs were used (in RStudio v. 2023.06.0 Build 421 © 2009–2023 Posit Software, PBC; R v. 4.4.3–2025-02-28 ucrt) to compare amongst the three segmentation methods in native space, separately for each dataset (HCP35, MAP35), and DV (Dice coefficient, AHD). The Holm-Bonferroni sequential correction method (Holm 1979) was employed to correct for the number of repeated-measures ANOVAs per dataset and metric (*n* = 22 for Morel, *n* = 16 for Marseille), as well as for the post-hoc pair-wise comparisons (*n* = 3) between segmentation methods. The Greenhouse-Geisser correction was used for the ANOVAs to adjust the degrees of freedom whenever the assumption of sphericity was violated.

### MNI-space analyses

Following the spatial transformation of all segmentations from native to MNI space, group-level probabilistic maps were generated, per segmentation of interest (*n* = 24; Supplementary Tables 2 and 3), method (*n* = 3), and dataset/group (*n* = 2). The proportion of each group that was assigned to a segmentation was determined at each voxel position (Najdenovska et al. 2018; Williams et al. 2024), for each of the three methods. A threshold of 0.25 was used to binarize the group-level probabilistic atlas (Williams et al. 2024). Dice coefficients and AHDs were derived for each group-level segmentation in MNI space for each method and dataset relative to the corresponding Morel or Marseille reference segmentation. The calculation of Dice and AHD metrics in both native and MNI space, as well as the generation of the group-level probabilistic atlases were conducted using SPM12 (v. 7771) in Matlab (v. 2023a Update 8–9.14.0.2891782) 64-bit).

### “Best” method per segmentation

**Fig. 1:**
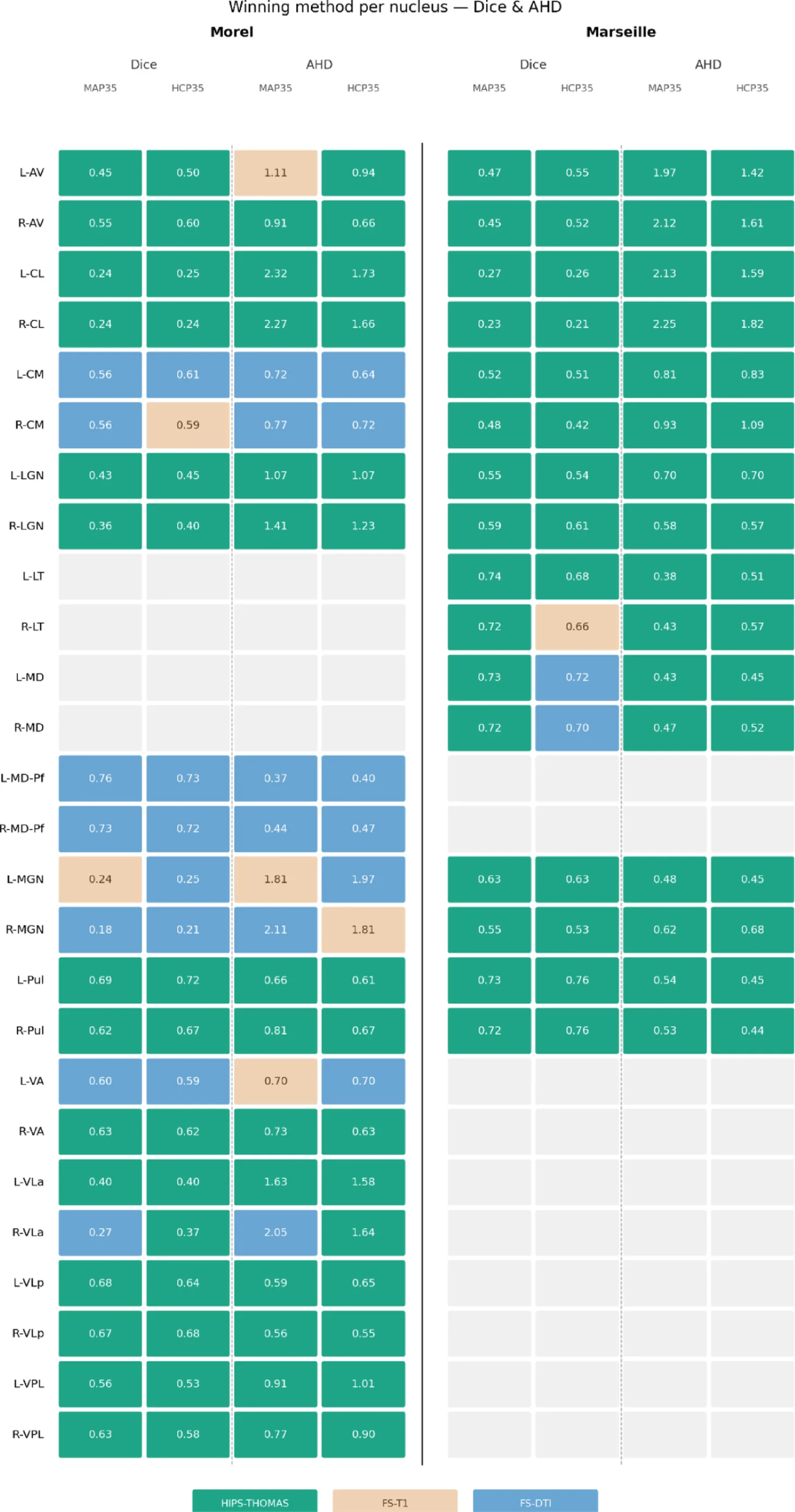
“Winner Map” of Dice coefficients and AHD for nuclei segmented with FS-T1, FS-DTI, and HIPS-THOMAS relative to Morel and Marseille reference segmentations (native space; see also Supplementary Figs. 1–4); AV: anteroventral nucleus; LT: lateral nuclei (Marseille); VA: ventral anterior nucleus; VLa: Ventrolateral anterior nucleus; VLp: Ventrolateral posterior nucleus; MD-Pf: mediodorsal-parafascicular nuclei; Pul: pulvinar nucleus; VPL: Ventral Posterolateral nucleus; CL: Centrolateral nucleus; CM: Centromedian nucleus; LGN: Lateral Geniculate Nucleus; MGN: Medial Geniculate Nucleus; L/R: Left, Right hemisphere; FS-T1: T_1_-based FreeSurfer segmentation (Iglesias et al. 2018); FS-DTI: FreeSurfer’s joint segmentation of thalamic nuclei from T1 scan and DTI (Tregidgo et al. 2023); (Vidal et al. 2024); AHD: Average Hausdorff Distance; HIPS-THOMAS: Thalamus Optimized Multi-atlas Segmentation using Histogram-based Polynomial Synthesis (Vidal et al. 2024); MAP35, HCP35: datasets with T1w-MRIs and diffusion MRI available; black diamond: mean; black horizontal line: median; Morel: Krauth-Morel atlas (Krauth et al. 2010); Marseille: the custom-made MNI version of the atlas of deep grey matter nuclei, part of the 7TAMIbrain dataset (Brun et al. 2022)

**Fig. 2:**
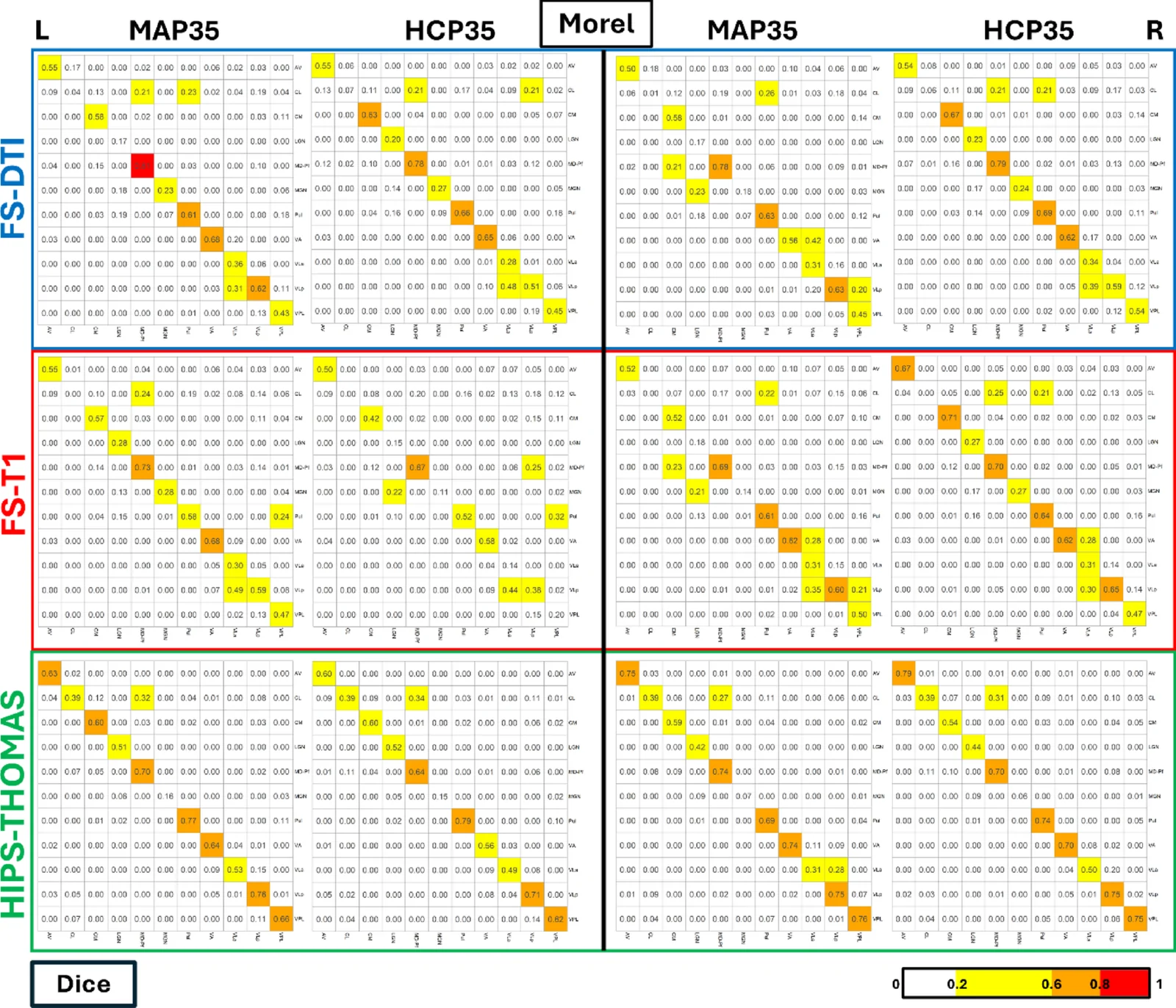
Group-level Dice coefficients for segmentations based on FS-T1, FS-DTI, and HIPS-THOMAS from MAP35, HCP35 (MNI space) relative to Morel reference segmentations (see also Supplementary Fig. 5 for AHD); AV: anteroventral nucleus; LT: lateral nuclei (Marseille); VA: ventral anterior nucleus; VLa: Ventrolateral anterior nucleus; VLp: Ventrolateral posterior nucleus; MD-Pf: mediodorsal-parafascicular nuclei; Pul: pulvinar nucleus; VPL: Ventral Posterolateral nucleus; CL: Centrolateral nucleus; CM: Centromedian nucleus; LGN: Lateral Geniculate Nucleus; MGN: Medial Geniculate Nucleus; L/R: Left, Right hemisphere; FS-T1: T_1_-based FreeSurfer segmentation (Iglesias et al. 2018); AHD: Average Hausdorff Distance; FS-DTI: FreeSurfer’s joint segmentation of thalamic nuclei from T1 scan and DTI (Tregidgo et al. 2023); HIPS-THOMAS: Thalamus Optimized Multi-atlas Segmentation using Histogram-based Polynomial Synthesis (Vidal et al. 2024); Morel: Krauth-Morel atlas (Krauth et al. 2010)

**Fig. 3:**
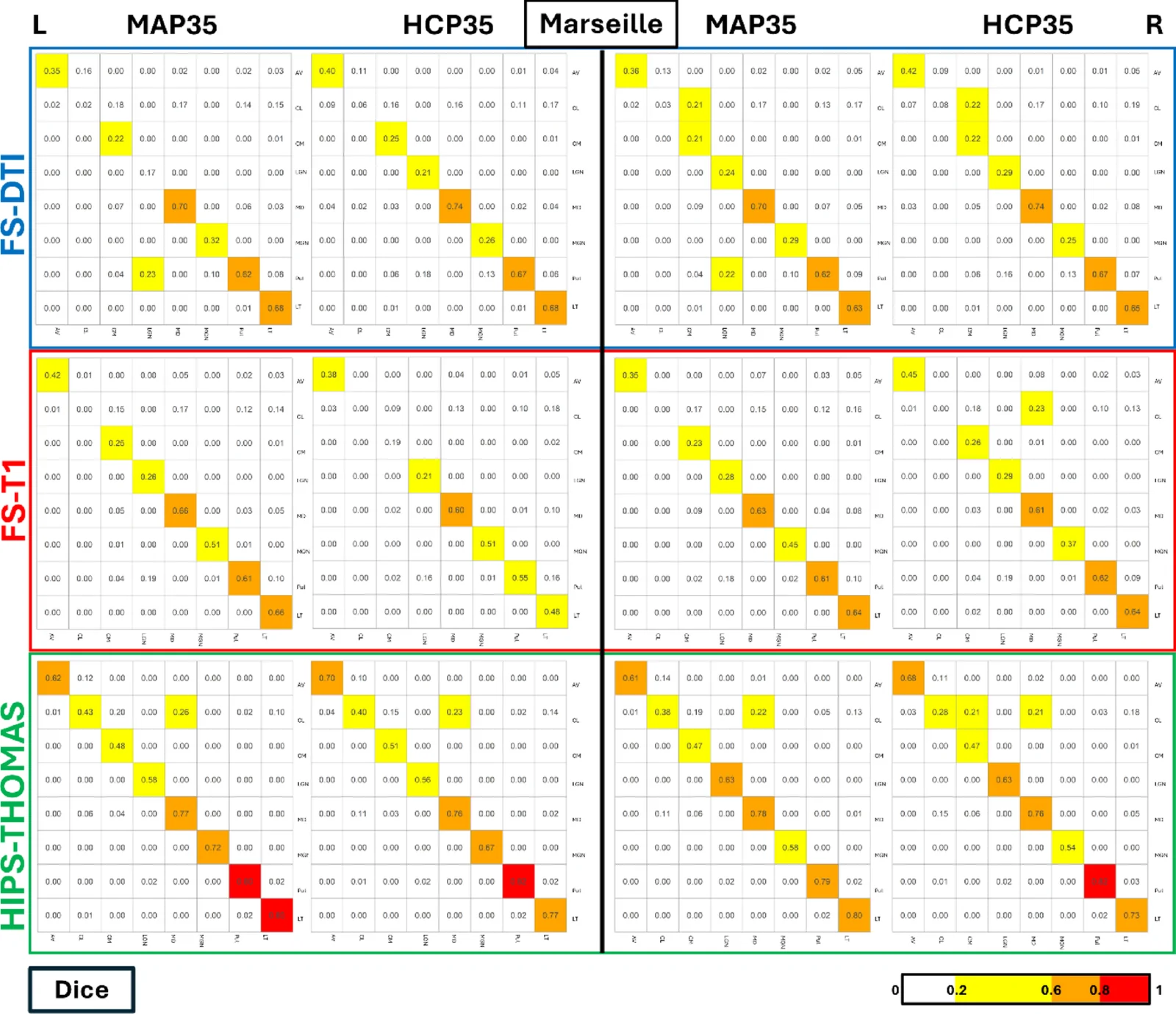
Group-level Dice coefficients for segmentations based on FS-T1, FS-DTI, and HIPS-THOMAS from MAP35, HCP35 (MNI space) relative to Marseille reference segmentations (see also Supplementary Fig. 6 for AHD); AV: anteroventral nucleus; LT: lateral nuclei (Marseille); VA: ventral anterior nucleus; VLa: Ventrolateral anterior nucleus; VLp: Ventrolateral posterior nucleus; MD-Pf: mediodorsal-parafascicular nuclei; Pul: pulvinar nucleus; VPL: Ventral Posterolateral nucleus; CL: Centrolateral nucleus; CM: Centromedian nucleus; LGN: Lateral Geniculate Nucleus; MGN: Medial Geniculate Nucleus; L/R: Left, Right hemisphere; AHD: Average Hausdorff Distance; FS-T1: T_1_-based FreeSurfer segmentation (Iglesias et al. 2018); FS-DTI: FreeSurfer’s joint segmentation of thalamic nuclei from T1 scan and DTI (Tregidgo et al. 2023); HIPS-THOMAS: Thalamus Optimized Multi-atlas Segmentation using Histogram-based Polynomial Synthesis (Vidal et al. 2024); Marseille: the custom-made MNI version of the atlas of deep grey matter nuclei, part of the 7TAMIbrain dataset (Brun et al. 2022)

**Fig. 4:**
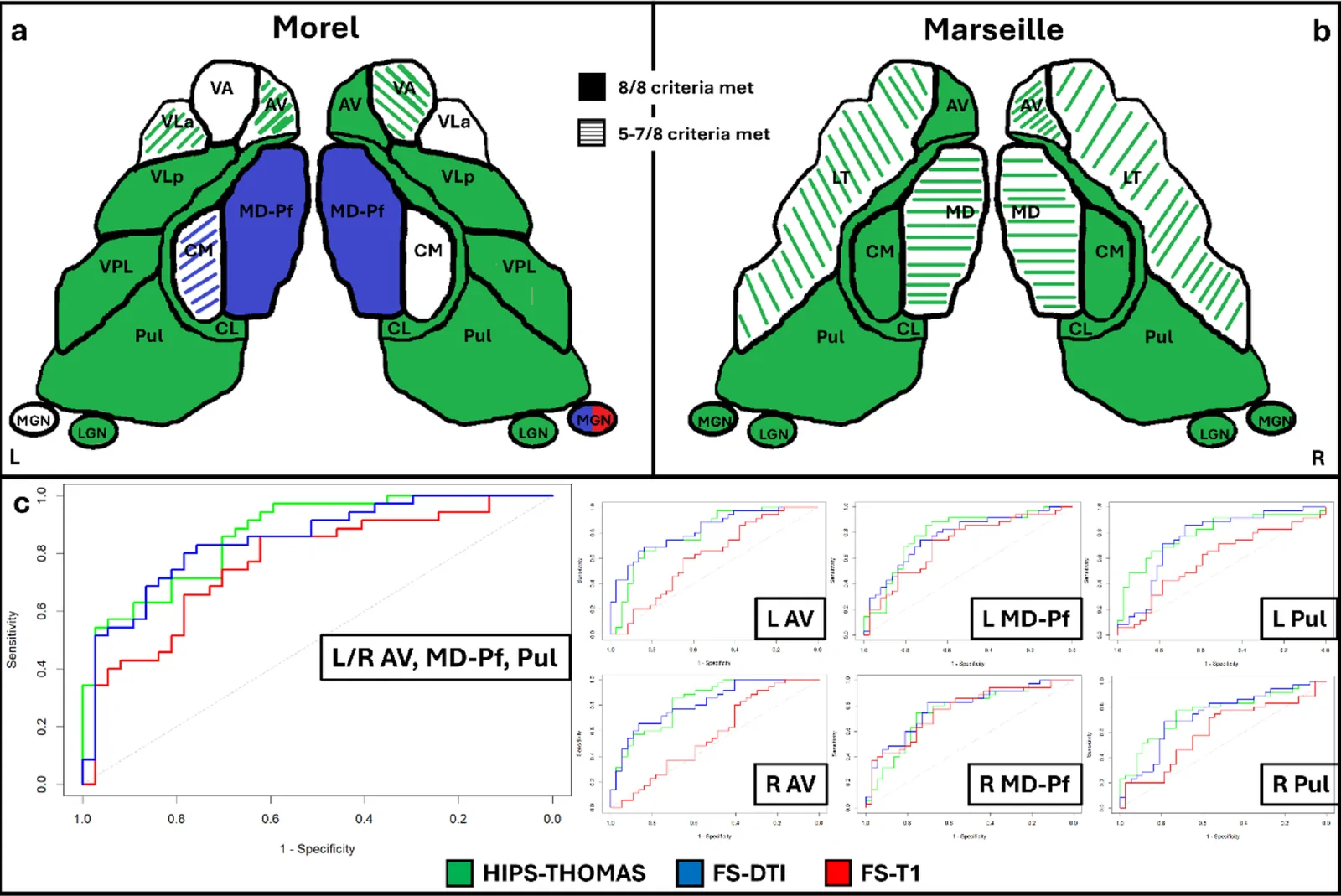
illustration of the best segmentation method for the 22 nuclei examined relative to (a) Morel and (b) Marseille; solid fill: best segmentation method; pattern fill (dashed lines): close-to-best method; no fill: no best or close-to-best method identified; c: ROC curves illustrating the three separate binomial logistic regression analyses (one per segmentation method), using all 6 segmentations of interest; c: ROC curves illustrating the 18 binomial logistic regression analyses conducted separately for each nucleus and segmentation method; AV: anteroventral nucleus; VA: ventral anterior nucleus; VLa: Ventrolateral anterior nucleus; LT: lateral nuclei (Marseille); VLp: Ventrolateral posterior nucleus; MD-Pf: mediodorsal-parafascicular nuclei; Pul: pulvinar nucleus; VPL: Ventral Posterolateral nucleus; CL: Centrolateral nucleus; CM: Centromedian nucleus; LGN: Lateral Geniculate Nucleus; MGN: Medial Geniculate Nucleus; L/R: Left, Right hemisphere; FS-T1: T_1_-based FreeSurfer segmentation (Iglesias et al. 2018); FS-DTI: FreeSurfer’s joint segmentation of thalamic nuclei from T1w-MRI and DTI (Tregidgo et al. 2023); HIPS-THOMAS: Thalamus Optimized Multi-atlas Segmentation using Histogram-based Polynomial Synthesis (Vidal et al. 2024); Morel: Krauth-Morel atlas (Krauth et al. 2010); Marseille: the custom-made MNI version of the atlas of deep grey matter nuclei, part of the 7TAMIbrain dataset (Brun et al. 2022)

A series (*n* = 8) of stringent criteria were applied to identify the “best” segmentation method, for each of the segmentations, separately for Morel and Marseille. A method (FS-DTI, FS-T1, HIPS-THOMAS) was identified as “the best” if it met all 8 of the criteria – i.e., if it surpassed the other two for both Dice (highest of all three) and AHD metrics (lowest of all three), in both native-space (statistically – Fig. 1, Supplementary Figs. 1–4, Supplementary Tables 4–7) and MNI space (numerically – Figs. 2 and 3, Supplementary Figs. 5 and 6), in both the HCP35 and MAP35 datasets. Two segmentation methods were identified as “joint-best” if they both consistently surpassed the third one on all the aforementioned criteria. Of the segmentations for which no best method was found to satisfy 8/8 criteria, the methods that satisfied > 4/8 and ≤ 7/8 criteria were identified (Fig. 4a, b).

### Receiver operating characteristic (ROC) analysis

ROC analyses were performed on the MAP35 dataset (35 healthy controls and 37 patients). Volumes were residualised against age, sex, and total intracranial volume (FS’s ‘eTIV’). Binomial logistic regression was used to quantify the ability of FS-T1, FS-DTI, and HIPS-THOMAS to discriminate between healthy controls and patients, on the basis of the 6 volumes (left/right mediodorsal-parafascicular, anteroventral, pulvinar). Note that these nuclei had shown volume reduction in HIPS-THOMAS and FS-DTI (Argyropoulos et al. 2026). Area under the curve (AUC) values were calculated for each of the three segmentation methods. All 6 nuclei were first included as predictors. However, to address collinearity issues and identify the performance of each method on each of these segmentations, these analyses were repeated separately per method and segmentation (*n* = 18). DeLong’s tests were used to compare amongst the AUCs of the three methods on each of those segmentations. P-values were corrected as above.

## Results

### Native-space analyses

HIPS-THOMAS outperformed both FS-T1 and FS-DT1 for 11 nuclei (right anteroventral, left/right centrolateral, left/right lateral geniculate, left/right pulvinar, left/right ventrolateral posterior, and left/right ventral posterolateral) consistently across datasets (HCP35, MAP35) and Dice and AHD metrics when using the Morel atlas. FS-DTI consistently outperformed FS-T1 and HIPS-THOMAS only on left/right mediodorsal-parafascicular. FS-T1 did not consistently outperform the other two for any segmentation. These findings were replicated in an even stronger fashion (in favour of HIPS-THOMAS) when using the Marseille atlas. Figure 1 provides a map of the winning method (highest Dice, lowest AHD) per nucleus, hemisphere, dataset, metric, and atlas. Supplementary Figs. 1–4 provide alternative illustrations (dumbbell plots, violin plots) of the same data. Inferential statistics are provided in Supplementary Tables 4–7.

### MNI-space analysis

Group-level Dice coefficients in MNI-space relative to Morel and Marseille atlases are tabulated in Figs. 2 and 3, respectively. The corresponding AHDs are tabulated in Supplementary Figs. 5and 6. Overall, segmentations had higher Dice coefficients and lower AHDs along the diagonal (same Morel and Marseille segmentations) across segmentation methods. However, several exceptions were noted, indicating failure to distinguish between segmentations. These selectively occurred for FS-DTI and FS-T1. They involved left/right centrolateral (notably, no voxels surpassed the probability threshold of 0.25 for the left/right centrolateral segmentations for FS-T1 in the HCP35 dataset), left/right lateral geniculate, left/right ventrolateral anterior, and left ventral posterolateral.

### Nucleus-wise “best” segmentation method

Overall, for both native-space and MNI-space analyses, for both the HCP35 and the MAP35 datasets, and for both atlases consulted, HIPS-THOMAS consistently showed a greater number of segmentations with a Dice coefficient of ≥ 0.6 (at least “substantial” agreement/overlap) and an AHD ≤ 1 (sufficiently small distance) than both FS-DTI and FS-T1. That was not consistently the case for FS-DTI relative to FS-T1 (Supplementary Table 8).

Against Morel, HIPS-THOMAS emerged as the “best” of the three methods for 12/22 nuclei (Fig. 4a). FS-DTI was the “best” for only 2/22 nuclei. FS-T1 did not surpass the other two methods in any segmentation (0/22). FS-T1 and FS-DTI were joint-best for the right medial geniculate. Ignoring HIPS-THOMAS, FS-DTI was consistently better (with respect to 8/8 criteria per segmentation) than FS-T1 only for 4/22 segmentations. HIPS-THOMAS was closest to being the best for left anteroventral (5/8 criteria satisfied) and left ventrolateral anterior (7/8), whereas FS-DTI was closest to being the best only for left centromedian (5/8). Against the Marseille atlas, FS-T1 and FS-DTI attained no “best” status for any of the 16 segmentations. HIPS-THOMAS was the best (8/8 criteria satisfied) for 11/16 nuclei, and closest to being the best for the remaining 5/16, satisfying 7/8 criteria for all 5 nuclei.

### ROC analysis

In three (one per segmentation method) logistic binomial regression analyses including all 6 volumes of interest as predictors (left/right anteroventral, mediodorsal-parafascicular, pulvinar), HIPS-THOMAS showed numerically the largest AUC (0.868) relative to FS-DTI (0.847) and FS-T1 (0.768; Fig. 4b). DeLong comparisons showed that FS-T1’s AUC was only marginally smaller than those of HIPS-THOMAS (z = 2.10, p-corr(3) = 0.107) and FS-DTI (z = 1.73, p-corr(3) = 0.166). AUC differences between HIPS-THOMAS and FS-DTI did not approach significance (z = 0.51, p-corr(3) = 0.608).

A series of 18 analyses (6 volumes * 3 methods) using each volume as a single predictor, showed that left and right mediodorsal-parafascicular volumes were significant (p-corr < 0.05) predictors of group membership (control vs. patient) for all three methods. However, left/right anteroventral and pulvinar volumes were significant predictors only for HIPS-THOMAS and FS-DTI (Table 1; Fig. 4c). DeLong comparisons (*n* = 18) between different methods on each of those 6 volumes, showed no difference amongst the three methods with respect to AUCs for mediodorsal-parafascicular, or between HIPS-THOMAS and FS-DTI on any of the 6 volumes. However, FS-T1’s AUCs were significantly or marginally smaller than those of HIPS-THOMAS and FS-DTI for left/right anteroventral and pulvinar (Table 2).

**Table 1:** Binomial logistic regression analyses, one per method and segmentation

Seg.	Hem.	Method	AUC	Coefficient	SE	z	*p*-corr(18)
AV	L	HIPS-THOMAS	0.80	1.28	0.35	3.71	0.003
		FS-DTI	0.82	1.68	0.42	4.01	0.001
		FS-T1	0.62	0.28	0.25	1.11	0.685
	R	HIPS-THOMAS	0.83	1.81	0.45	4.00	0.001
		FS-DTI	0.82	1.74	0.45	3.85	0.002
		FS-T1	0.57	0.22	0.24	0.89	0.685
MD-Pf	L	HIPS-THOMAS	0.79	1.27	0.35	3.63	0.004
		FS-DTI	0.77	1.26	0.37	3.40	0.007
		FS-T1	0.71	0.82	0.28	2.90	0.019
	R	HIPS-THOMAS	0.76	1.15	0.35	3.28	0.008
		FS-DTI	0.78	1.37	0.39	3.48	0.006
		FS-T1	0.76	1.16	0.34	3.43	0.007
Pul	L	HIPS-THOMAS	0.80	1.28	0.35	3.67	0.003
		FS-DTI	0.76	1.10	0.33	3.37	0.007
		FS-T1	0.59	0.29	0.24	1.21	0.685
	R	HIPS-THOMAS	0.76	1.26	0.38	3.30	0.008
		FS-DTI	0.73	1.13	0.37	3.05	0.014
		FS-T1	0.61	0.36	0.25	1.44	0.599

p-corr(18): the p-value was adjusted to correct for the number of multiple comparisons made (*n* = 18), using the Holm-Bonferroni sequential correction method (Holm 1979)

AV: anteroventral nucleus; MD-Pf: mediodorsal-parafascicular nucleus; Pul: pulvinar nucleus; L/R: Left, Right hemisphere; FS-T1: T_1_-based FreeSurfer segmentation (Iglesias et al. 2018); FS-DTI: FreeSurfer’s joint segmentation of thalamic nuclei from T1 scan and DTI (Tregidgo et al. 2023); HIPS-THOMAS: Thalamus Optimized Multi-atlas Segmentation using Histogram-based Polynomial Synthesis (Vidal et al. 2024); Seg.: Segmentation; Hem.: Hemisphere; SE: Standard Error; AUC: Area Under the Curve

**Table 2:** DeLong comparisons of segmentation methods on AUCs for L/R AV, MD-Pf, and Pul

Seg.	Hem.	Method-1	Method-2	z	*p*-corr(18)
AV	L	HIPS-THOMAS	FS-DTI	-0.42	> 0.999
			FS-T1	2.75	0.077
		FS-DTI		3.63	0.005
	R	HIPS-THOMAS	FS-DTI	0.24	> 0.999
			FS-T1	4.12	< 0.001
		FS-DTI		3.32	0.013
MD-Pf	L	HIPS-THOMAS	FS-DTI	0.40	> 0.999
			FS-T1	1.39	
		FS-DTI		1.33	
	R	HIPS-THOMAS	FS-DTI	-0.57	
			FS-T1	-0.16	
		FS-DTI		0.47	
Pul	L	HIPS-THOMAS	FS-DTI	0.91	
			FS-T1	3.53	0.007
		FS-DTI		2.47	0.164
	R	HIPS-THOMAS	FS-DTI	0.68	> 0.999
			FS-T1	2.88	0.056
		FS-DTI		2.12	0.374

p-corr(18): the p-value was adjusted to correct for the number of multiple comparisons made (*n* = 18), using the Holm-Bonferroni sequential correction method (Holm 1979)

AV: anteroventral segmentation; MD-Pf: mediodorsal-parafascicular segmentation; Pul: pulvinar segmentation; L/R: Left, Right hemisphere; FS-T1: T_1_-based FreeSurfer segmentation (Iglesias et al. 2018); FS-DTI: FreeSurfer’s joint segmentation of thalamic nuclei from T1 scan and DTI (Tregidgo et al. 2023); HIPS-THOMAS: Thalamus Optimized Multi-atlas Segmentation using Histogram-based Polynomial Synthesis (Vidal et al. 2024); Seg.: Segmentation; Hem.: Hemisphere

## Discussion

Recent developments in automated segmentation of the human thalamus have substantially improved our understanding of the integrity, activity, and connectivity of individual thalamic nuclei in health and disease. Although there are several methods available for segmentation, studies comparing their accuracy and reliability are scant. Relying on suboptimal segmentation methods and cherry-picking amongst alternatives may compromise the efficacy of neuromodulatory interventions targeting the thalamus, as well as the replication of comparative neuroanatomical studies and clinical cognitive neuroimaging research.

Here, we used two independent datasets of healthy adults with T1w-MRI and diffusion MRI data to compare amongst three state-of-the art structural MRI-based thalamic segmentation methods: the more widely used one (FS-T1 (Iglesias et al. 2018), along with two more recently developed ones: FS-DTI (Tregidgo et al. 2023), and HIPS-THOMAS (Vidal et al. 2024). Unlike the other two methods, FS-DTI leverages information from both T1w-MRI and diffusion MRI. Whereas HIPS-THOMAS has been shown to surpass FS-T1 (Williams et al. 2024), it has not been compared with FS-DTI, and we are also unaware of the extent to which FS-DTI enhances thalamic segmentation relative to FS-T1. Moreover, some of the work that has highlighted limitations in FS-T1 (Williams et al. 2022, 2024) has relied on the (spatially normalised in MNI space) Morel atlas (Krauth et al. 2010; Jakab et al. 2012) – the use of which as such is not unproblematic (Brun et al. 2022). It is thus important to examine whether comparisons between segmentation methods can be replicated with alternative reference atlases, other than Morel.

Surprisingly, despite relying exclusively on structural T1w-MRI inputs, HIPS-THOMAS consistently outperformed, not just FS-T1 (dovetailing with earlier observations (Williams et al. 2024), but also FS-DTI, and did so irrespective of the comparison atlas used - Marseille or Morel. Against Morel, HIPS-THOMAS outperformed the other two on 12/22 nuclei and outperformed them in a less consistent fashion in another 2. FS-DTI consistently outperformed FS-T1 in only 4/22 segmentations when using Morel, and in 3/16 when using Marseille. Using the Marseille atlas, HIPS-THOMAS outperformed the other two on all 16 segmentations – consistently (8/8 criteria met) on 11/16, and less consistently (7/8 criteria met) on the remaining 5/16. The improved performance of FS-DTI over FS-T1 has been attributed to its additional incorporation of fractional anisotropy FA maps which can help discriminate WM-rich areas such as the internal lamina and the white-matter tracts outside the ventral thalamic nuclei against the grey-matter dominant thalamic tissue. The use of WM-nulled contrast achieves the same end but using just structural imaging, presumably contributing to the superior performance of HIPS-THOMAS. This is further corroborated by the fact that FS-DTI is much closer to HIPS-THOMAS than FS-T1 (Supplementary Figs. 1 and 3).

When using Morel, the only segmentation in which FS-DTI outperformed HIPS-THOMAS and FS-T1 was the mediodorsal-parafascicular, bilaterally, and less consistently for left centromedian. Even so, across datasets and analyses (mean values for native space, group MNI values), the HIPS-THOMAS mediodorsal-parafascicular segmentations showed Dice coefficients that had a minimum of 0.58, and AHDs with a maximum of 0.96. Moreover, in the AUC analyses, the mediodorsal-parafascicular volumes from HIPS-THOMAS remained significant predictors of group membership and did not differ in this regard from FS-T1 or FS-DTI. However, HIPS-THOMAS did not show these limitations when using the Marseille atlas. Given the inconsistencies between Morel and Marseille on mediodorsal-parafascicular, centromedian, and medial geniculate, it would be important to approach the findings reported in (Williams et al. 2024) on these segmentations with caution. In that paper, FS-T1 was the best segmentation method for the left mediodorsal-parafascicular, the right centromedian (and joint best with HIPS-THOMAS on the left centromedian), and for left medial geniculate. Whether this should be attributed to the limitations of Morel is outside the scope of this investigation. Moreover, given that HIPS-THOMAS segmentations are substantially fewer than those of FS-T1/FS-DTI, we have confined our analyses to segmentations that are common across all three of the methods. Our comments on the improvement that FS-DTI makes relative to FS-T1 thus pertain to only those 22 nuclei in question.

Even so, FS-T1’s failure to discriminate between healthy controls and patients on the grounds of anteroventral and pulvinar volumes is strongly consistent with (Williams et al. 2024), wherein FS-T1 performed quite poorly in discriminating healthy controls from those with mild cognitive impairment and Alzheimer’s disease for anteroventral and pulvinar (note that FS-DTI was not part of these comparisons). Given that automated thalamic segmentation may be affected by condition-specific abnormalities in neurological patients (e.g., ventricular enlargement, white matter damage in tracts connecting the thalamus (Segobin et al. 2024), comparisons of these methods in different conditions are particularly important. Along with those of (Williams et al. 2024), our AUC analyses thus highlight the danger of false negatives in studies that have relied on FS-T1.

Arguably, low-quality segmentations may also result in false positives. Several studies using FS-T1 have disclosed volume reduction in segmentations that we and (Williams et al. 2024) have shown to have poor segmentation accuracy. Examples include the reduction observed (among others) in anteroventral, lateral geniculate, ventral anterior, ventrolateral anterior, ventrolateral posterior, and ventral posterolateral in major depressive disorder (Zhang et al. 2024), in pulvinar for paediatric bipolar disorder (Gao et al. 2025), in pulvinar and lateral geniculate for prenatal alcohol exposure (Nakhid et al. 2023), in pulvinar for remitting-relapsing multiple sclerosis (Trufanov et al. 2021), in pulvinar and lateral geniculate for schizophrenia spectrum disorders and bipolar disorders (Mørch-Johnsen et al. 2023). Likewise, caution would be advisable in interpreting the findings of tractography studies using FS-T1 to segment the aforementioned nuclei - e.g., lateral geniculate and ventrolateral posterior in (Liu et al. 2022).

To potentially minimise measurement error along with the number of statistical tests, several studies (e.g. (Bergsland et al. 2021; Pardilla-Delgado et al. 2021; Tung et al. 2022; McKenna et al. 2022; Shah et al. 2024; Thalhammer et al. 2024)) that employ FS-T1, group thalamic nuclei into subregions, based on their spatial arrangement (e.g., the anteroventral is assigned to the anterior region, the laterodorsal and lateral posterior nuclei assigned to a lateral region, the lateral geniculate, medial geniculate, limitans-suprageniculate, anterior pulvinar, medial pulvinar, lateral pulvinar, and inferior pulvinar may be assigned to a posterior region), or their functional anatomy (e.g., the anteroventral may be its own group, whereas the ventral anterior, ventral anterior magnocellular, ventrolateral anterior, and ventrolateral posterior may be assigned to a ‘Motor’ group). Apart from impeding the localisation of the effects of interest, this approach ignores the substantial variability across these composite ROIs with respect to their volume, the number of nuclei each of those comprises, and, as shown here, their segmentation quality.

Finally, the fact that HIPS-THOMAS was as good as FS-DTI in distinguishing between healthy controls and patients on the basis of the volumes of nuclei of interest is noteworthy. Substantial resources are involved in collecting the data necessary (diffusion MRI) and completing the additional preprocessing stages to derive the FS-DTI segmentations. The use of FS-DTI would be further impeded by the fact that several open MRI databases may contain T1w-MRI, but not high-quality diffusion MRI data. On the other hand, HIPS-THOMAS holds the additional advantage of catering for the segmentation of both thalamic nuclei and other subcortical structures with much shorter computational time and within a single unified package - see discussion in (Saranathan et al. 2025).

Our study had some limitations. We limited our comparisons to state-of-the-art structural MRI-based segmentation methods, and did not consider diffusion-based or fMRI-based methods (Battistella et al. 2017; van Oort et al. 2018). We used Morel as one of the “silver” standards for comparisons, especially for Dice and AHD calculations. Both HIPS-THOMAS and Freesurfer follow Morel’s Anglo-Saxon nomenclature and have compared their segmentations against Morel in their methodology papers (Iglesias et al. 2018; Tregidgo et al. 2023; Vidal et al. 2024). To address possible shortcomings in the translation of histological to MRI space, we included an additional atlas (Marseille) which is driven more by MRI contrast (Brun et al. 2022). This atlas was developed using the Morel atlas as a visual guide, much like THOMAS, using the same ontology and boundaries and hence consistent. Future work could use newer atlases, such as the Allen-Brain (Jones et al. 2009), but prior work on harmonizing the ontology across diverse atlases should occur before this would yield meaningful results. Mai and Majtanik (2019) have shown that at a regional level that Morel and Allen Brain show a fairly high degree of concordance, but this needs to be investigated at thalamic nuclear level.

Given the above, we urge the community to exercise caution whilst using FS-T1 and depending on the availability of diffusion MRI data, corroborate findings with FS-DTI. HIPS-THOMAS can provide a viable and accurate alternative when only structural T1 MRI data is available. Replicating FS-T1-based analyses in already published studies with HIPS-THOMAS and FS-DTI would also be instructive, for the purposes of minimising false positives/negatives.

## Key points

We compared, for the first time, three key methods for automatic segmentation of human thalamic nuclei: HIPS-THOMAS, FreeSurfer, and its recent, diffusion-based update. HIPS-THOMAS surpassed FreeSurfer methods in healthy adults, while the standard FreeSurfer method underperformed in distinguishing between healthy controls and patients with hippocampal damage.

## Supplementary Information

Below is the link to the electronic supplementary material.

Supplementary Material 1

## Data Availability

Demographic and volumetric data are publicly available on https://osf.io/8bp64/. Bash, Matlab, and R scripts are also available there. HCP35 (MGH HCP Adult Diffusion Dataset ID: “MGH_DIFF”): https://db.humanconnectome.org/data/projects/MGH_DIFF. Data collection and sharing for this project was provided by the Human Connectome Project (HCP; Principal Investigators: Bruce Rosen, M.D., Ph.D., Martinos Center at Massachusetts General Hospital; Arthur W. Toga, Ph.D., University of Southern California; Van J. Weeden, MD, Martinos Center at Massachusetts General Hospital). HCP funding was provided by the National Institute of Dental and Craniofacial Research (NIDCR), the National Institute of Mental Health (NIMH), and the National Institute of Neurological Disorders and Stroke (NINDS). HCP data are disseminated by the Laboratory of Neuro Imaging at the University of Southern California. HCP is the result of efforts of co-investigators from the University of Southern California, Martinos Center for Biomedical Imaging at Massachusetts General Hospital (MGH), Washington University, and the University of Minnesota. FS-T1: FreeSurfer segmentation was Conducted following the instructions provided in https://freesurfer.net/fswiki/ThalamicNuclei). HIPS-THOMAS: Details for running HIPS-THOMAS using Docker are available on Github (HIPS-THOMAS https://github.com/thalamicseg/hipsthomasdocker); the Docker images are available on Docker Hub (https://hub.docker.com/u/anagrammarian). FS-DTI: details on its use can be found in https://surfer.nmr.mgh.harvard.edu/fswiki/ThalamicNucleiDTI. We used the updated model (`thalseg_1.1.h5`) made available https://ftp.nmr.mgh.harvard.edu/pub/dist/freesurfer/thalseg/ThalsegPatch_1.1.tgz) (validated against additional datasets) which we called with the “—model” option. Improvements to the segmentation script were downloaded from https://github.com/freesurfer/freesurfer/blob/dev/mri_segment_thalamic_nuclei_dti_cnn/mri_segment_thalamic_nuclei_dti_cnn and were used to replace the release version, in order to prevent occurrences of false positive thalamic segmentations; “Morel”: Krauth-Morel atlas, available on https://zenodo.org/records/13918589; “Marseille”: 7TAMIbrainDGN, available on https://github.com/arnaudletroter/7TAMIBrain.
