# Supplementary material for: Toward reliable thalamic segmentation: an evaluation of automated methods for structural MRI
Source: Brain Struct Funct. 2026 Jul 18;231(7):102. doi: 10.1007/s00429-026-03163-z (PMC13380577; doi:10.1007/s00429-026-03163-z)
Supplement: Supplementary file 1 — Supplementary Material 1 [file 429_2026_3163_MOESM1_ESM.docx]

# Supplementary Information

- **article title:** Toward Reliable Thalamic Segmentation: a rigorous evaluation of automated methods for structural MRI
- **journal name:** Brain Structure and Function
- **author names:** Georgios P. D. Argyropoulos, Christopher R. Butler, Manojkumar Saranathan
- **affiliation and e-mail address of the corresponding author:** Memory Research Group, Nuffield Department of Clinical Neurosciences, University of Oxford, Oxford, United Kingdom; Division of Psychology, Faculty of Natural Sciences, University of Stirling, Scotland, United Kingdom. Correspondence: Georgios P. D. Argyropoulos, Division of Psychology, Faculty of Natural Sciences, University of Stirling, Stirling FK9 4LA, UK. Email: [georgios.argyropoulos@stir.ac.uk](mailto:georgios.argyropoulos@stir.ac.uk)

## Supplementary Methods – Segmented Nuclei per Method

**FS-T1:** The segmented nuclei comprise the anteroventral (AV), the centrolateral (CL), centromedian (CM), central medial, lateral geniculate (LGN), lateral posterior, laterodorsal, limitans-suprageniculate, medial geniculate (MGN), mediodorsal lateral-parvocellular (MDl), mediodorsal medial-magnocellular (MDm), reuniens-medial ventral, paracentral, parafascicular (Pf), paratenial, pulvinar anterior (PuA), pulvinar inferior (PuI), pulvinar lateral (PuL), pulvinar medial (PuM), ventral anterior (VA), ventral anterior magnocellular (VAmc), ventral lateral anterior (VLa), ventral lateral posterior (VLp), ventromedial, ventral posterolateral nuclei (VPL).

**FS-DTI:** The segmented nuclei are the same as those of FS-T1, with the following exceptions: two segmentations are generated for PuM: a medial segment, PuMm, and a lateral one (PuMl). The paracentral, ventromedial, and paratenial segmentations are not generated, as they do not appear in all of the training examples for the FS-DTI’s CNN, so these are excluded to improve the robustness of the network (<https://surfer.nmr.mgh.harvard.edu/fswiki/ThalamicNucleiDTI>).

**HIPS-THOMAS:** The segmented nuclei comprise the AV, VA, VLa, VLp, VPL, Pul, LGN, MGN, CM, MD-Pf, and habenular nuclei as well as CL.

## Supplementary Table 1

| **Dataset** | **Subject** | **Total thalamic volume (n vox)** | | |
| --- | --- | --- | --- | --- |
|  |  | **FS-DTI** | **FS-T1** | **HIPS-THOMAS** |
| HCP35 | mgh_1001 | 10753 | 12213 | 9845 |
| HCP35 | mgh_1002 | 11830 | 12818 | 10495 |
| HCP35 | mgh_1003 | 12958 | 14627 | 11598 |
| HCP35 | mgh_1004 | 13451 | 12453 | 11470 |
| HCP35 | mgh_1005 | 10551 | 11952 | 10063 |
| HCP35 | mgh_1006 | 13679 | 12926 | 11523 |
| HCP35 | mgh_1007 | 12453 | 16887 | 13117 |
| HCP35 | mgh_1008 | 13937 | 13333 | 11608 |
| HCP35 | mgh_1009 | 13456 | 13674 | 11892 |
| HCP35 | mgh_1010 | 13094 | 12317 | 10945 |
| HCP35 | mgh_1011 | 11961 | 12720 | 10850 |
| HCP35 | mgh_1012 | 13439 | 12630 | 10835 |
| HCP35 | mgh_1013 | 13073 | 13128 | 10906 |
| HCP35 | mgh_1014 | 15556 | 14239 | 11815 |
| HCP35 | mgh_1015 | 11973 | 13966 | 10926 |
| HCP35 | mgh_1016 | 14451 | 13179 | 11845 |
| HCP35 | mgh_1017 | 11372 | 11709 | 9745 |
| HCP35 | mgh_1018 | 15352 | 14261 | 12199 |
| HCP35 | mgh_1019 | 12411 | 13142 | 11566 |
| HCP35 | mgh_1020 | 2957 | 14504 | 11481 |
| HCP35 | mgh_1021 | 11739 | 11698 | 9825 |
| HCP35 | mgh_1022 | 14479 | 14551 | 13724 |
| HCP35 | mgh_1023 | 10332 | 11579 | 10104 |
| HCP35 | mgh_1024 | 11750 | 11920 | 10576 |
| HCP35 | mgh_1025 | 13467 | 14743 | 11742 |
| HCP35 | mgh_1026 | 12627 | 12487 | 11265 |
| HCP35 | mgh_1027 | 11716 | 14735 | 10817 |
| HCP35 | mgh_1028 | 10519 | 10779 | 8560 |
| HCP35 | mgh_1029 | 14338 | 16234 | 13018 |
| HCP35 | mgh_1030 | 12626 | 13268 | 11647 |
| HCP35 | mgh_1031 | 12228 | 15345 | 11887 |
| HCP35 | mgh_1032 | 10804 | 10185 | 9370 |
| HCP35 | mgh_1033 | 12473 | 12498 | 11285 |
| HCP35 | mgh_1034 | 15600 | 16408 | 13747 |
| HCP35 | mgh_1035 | n/a | n/a | n/a |
| MAP35 | C1 | 15611 | 14490 | 12833 |
| MAP35 | C2 | 14720 | 13574 | 12220 |
| MAP35 | C3 | 10807 | 12314 | 9900 |
| MAP35 | C4 | 11257 | 11782 | 9341 |
| MAP35 | C5 | 13516 | 14023 | 11092 |
| MAP35 | C6 | 10117 | 12078 | 8905 |
| MAP35 | C7 | 11986 | 12294 | 9665 |
| MAP35 | C8 | 14025 | 12616 | 11455 |
| MAP35 | C9 | 10186 | 11062 | 9175 |
| MAP35 | C10 | 11870 | 12240 | 9568 |
| MAP35 | C11 | 11504 | 13056 | 9933 |
| MAP35 | C12 | 13076 | 13087 | 10686 |
| MAP35 | C13 | 14485 | 14309 | 11702 |
| MAP35 | C14 | 15961 | 15396 | 12791 |
| MAP35 | C15 | 10829 | 11950 | 9931 |
| MAP35 | C16 | 11288 | 12052 | 9929 |
| MAP35 | C17 | 10627 | 11994 | 9614 |
| MAP35 | C18 | 10955 | 12277 | 9545 |
| MAP35 | C19 | 10953 | 11494 | 9045 |
| MAP35 | C20 | 11747 | 13213 | 10485 |
| MAP35 | C21 | 11105 | 13234 | 9779 |
| MAP35 | C22 | 13600 | 13646 | 11742 |
| MAP35 | C23 | 10618 | 11697 | 9264 |
| MAP35 | C24 | 11075 | 12348 | 9824 |
| MAP35 | C25 | 13763 | 14661 | 12251 |
| MAP35 | C26 | 13154 | 14967 | 12082 |
| MAP35 | C27 | 13348 | 14196 | 11572 |
| MAP35 | C28 | 13340 | 14358 | 11519 |
| MAP35 | C29 | 12838 | 12704 | 10412 |
| MAP35 | C30 | 14628 | 14575 | 12673 |
| MAP35 | C31 | 15121 | 14064 | 11956 |
| MAP35 | C32 | 9524 | 9732 | 8393 |
| MAP35 | C33 | 10954 | 11380 | 10142 |
| MAP35 | C34 | 14338 | 12768 | 11535 |
| MAP35 | C35 | 15459 | 14741 | 12612 |

Total thalamic volumes derived from the three segmentation methods. FS-DTI: FreeSurfer’s joint segmentation of thalamic nuclei from T1 scan and DTI (Tregidgo et al., 2023); HIPS-THOMAS: Thalamus Optimized Multi-atlas Segmentation using Histogram-based Polynomial Synthesis (Vidal et al., 2024); MAP35, HCP35: datasets with T1-weighted MRIs and diffusion MRI available

## Supplementary Table 2

| **HIPS-THOMAS** | **FS-T1** | **FS-DTI** | **Krauth-Morel** |
| --- | --- | --- | --- |
| AV | AV | AV | AV |
| CL | CL | CL | CL |
| CM | CM | CM | CM |
| LGN | LGN | LGN | LGNmc+LGNpc |
| MD-Pf | MDl+ MDm + Pf | MDl+ MDm + Pf | Pf + sPf + MDmc + MDpc |
| MGN | MGN | MGN | MGN |
| Pul | PuA+PuI+PuL+PuM | PuA+PuI+PuL+PuMl+PuMm | PuA+PuI+PuL+PuM |
| VA | VAmc + VApc | VAmc + VApc | VAmc + VApc |
| VLa | VLa | VLa | VLa |
| VLp | VLp | VLp | VLpd + VLpv |
| VPL | VPL | VPL | VPLa+VPLp |

Method for combining FreeSurfer and Krauth-Morel nuclei to match the Morel nomenclature used by THOMAS, producing a unified space for thalamic segmentation comparison; **key:** AV: anteroventral nucleus; VA: ventral anterior nucleus; VLa: Ventrolateral anterior nucleus; VLp: Ventrolateral posterior nucleus; MD-Pf: mediodorsal-parafascicular nuclei; Pul: pulvinar nucleus; VPL: Ventral Posterolateral nucleus; CL: Centrolateral nucleus; CM: Centromedian nucleus; LGN: Lateral Geniculate Nucleus; MGN: Medial Geniculate Nucleus; MDl: mediodorsal nucleus – lateral portion; MDm: mediodorsal nucleus – medial portion; MDmc: mediodorsal nucleus – magnocellular portion; MDpc: mediodorsal nucleus – parvocellular portion; Pf: parafascicular nucleus; PuA: anterior pulvinar; PuI: inferior pulvinar; PuL: lateral pulvinar; PuM: medial pulvinar; PuMl: medial pulvinar (lateral segment for FS-DTI); PuMm: medial pulvinar (medial segment for FS-DTI) VAmc: ventral anterior magnocellular; VApc: ventral anterior parvocellular; LGNmc: lateral geniculate magnocellular; LGNpc: lateral geniculate parvocellular; sPf: subparafascicular; VLpd: ventrolateral posterior nucleus (dorsal) ; VLpv: ventrolateral posterior nucleus (ventral); VPLa: ventral posterolateral nucleus (anterior); VPLp: ventral posterolateral nucleus (posterior); L/R: Left, Right hemisphere; FS-T1: T_1_-based FreeSurfer segmentation (Iglesias et al., 2018); FS-DTI: FreeSurfer’s joint segmentation of thalamic nuclei from T1 scan and DTI (Tregidgo et al., 2023); HIPS-THOMAS: Thalamus Optimized Multi-atlas Segmentation using Histogram-based Polynomial Synthesis (Vidal et al., 2024); MAP35, HCP35: datasets with T1-weighted MRIs and diffusion MRI available; Morel: Krauth-Morel atlas (Krauth et al., 2010)

## Supplementary Table 3

| **Marseille** | **Krauth-Morel** | **HIPS-THOMAS** | **FS-T1** | **FS-DTI** |
| --- | --- | --- | --- | --- |
| AV | AV + AM + AD + LD | AV | AV | AV |
| CL | CL | CL | CL | CL |
| CM | CM | CM | CM | CM |
| LGN | LGNmc + LGNpc | LGN | LGN | LGN |
| MD | MDmc + MDpc | MD-Pf | MDl+ MDm + Pf | MDl+ MDm + Pf |
| MGN | MGN | MGN | MGN | MGN |
| Pu+PuA | (PuM + PuI + PuL)+ PuA | Pul | PuA+PuI+PuL+PuM | PuA+PuI+PuL+PuMl+PuMm |
| LT | VPM+VPLa+VPLp+VLa+VLpd+VLpv+  VM+VAmc+VApc+VPI | VPL+VLa+VLp+VA | VPL+VLa+VLp+VAmc+VApc | VPL+VLa+VLp+VAmc+VApc |

Method for combining FreeSurfer and HIPS-THOMAS nuclei to match the segmentations of interest in the Marseille atlas (Brun et al., 2022); **key:** AD: anterodorsal nucleus; AM: anteromedial nucleus; AV: anterior segmentation (Marseille atlas) or anteroventral nucleus (all other columns); CL: Centrolateral nucleus; CM: Centromedian nucleus; FS-DTI: FreeSurfer’s joint segmentation of thalamic nuclei from T1 scan and DTI (Tregidgo et al., 2023); FS-T1: T_1_-based FreeSurfer segmentation (Iglesias et al., 2018); HIPS-THOMAS: Thalamus Optimized Multi-atlas Segmentation using Histogram-based Polynomial Synthesis (Vidal et al., 2024); Morel: Krauth-Morel atlas (Krauth et al., 2010); L/R: Left, Right hemisphere; LD: laterodorsal nucleus; LGN: Lateral Geniculate Nucleus; LGNmc: lateral geniculate magnocellular nucleus; LGNpc: lateral geniculate parvocellular nucleus; LT: lateral nuclei (Marseille atlas); MAP35, HCP35: datasets with T1-weighted MRIs and diffusion MRI available; MD-Pf: mediodorsal-parafascicular nuclei; MD: mediodorsal nucleus (Marseille atlas); MDl: mediodorsal nucleus – lateral portion; MDm: mediodorsal nucleus – medial portion; MDmc: mediodorsal nucleus – magnocellular portion; MDpc: mediodorsal nucleus – parvocellular portion; MGN: Medial Geniculate Nucleus; Pf: parafascicular nucleus; Pu: Pulvinar (Marseille atlas), including PuM, PuI, and PuL nucleus; PuA: anterior pulvinar nucleus; PuI: inferior pulvinar nucleus; PuL: lateral pulvinar nucleus; Pul: pulvinar nucleus; Pul: Pulvinar nucleus, in all other columns; PuM: medial pulvinar nucleus; PuMl: medial pulvinar nucleus (lateral segment for FS-DTI); PuMm: medial pulvinar nucleus (medial segment for FS-DTI); sPf: subparafascicular nucleus; VA: ventral anterior nucleus; VAmc: ventral anterior magnocellular; VApc: ventral anterior parvocellular; VLa: Ventrolateral anterior nucleus; VLp: Ventrolateral posterior nucleus; VLpd: ventrolateral posterior nucleus (dorsal); VLpv: ventrolateral posterior nucleus (ventral); VM: ventromedial VPL: Ventral Posterolateral nucleus; VPLa: ventral posterolateral nucleus (anterior); VPLp: ventral posterolateral nucleus (posterior); VPM: ventral posteromedial nucleus.

## Supplementary Table 4

| **Seg.** | **Hem.** | **HCP35** | | | | | | | | | **MAP35** | | | | | | | | |
| --- | --- | --- | --- | --- | --- | --- | --- | --- | --- | --- | --- | --- | --- | --- | --- | --- | --- | --- | --- |
|  |  | **F** | **p-corr(22)** | **ges** | **HIPS-THOMAS vs. FS-T1** | | **HIPS-THOMAS vs. FS-DTI** | | **FS-T1 vs. FS-DTI** | | **F** | **p-corr(22)** | **ges** | **HIPS-THOMAS vs. FS-T1** | | **HIPS-THOMAS vs. FS-DTI** | | **FS-T1 vs. FS-DTI** | |
|  |  |  |  |  | **t** | **p-corr(3)** | **t** | **p-corr(3)** | **t** | **p-corr(3)** |  |  |  | **t** | **p-corr(3)** | **t** | **p-corr(3)** | **t** | **p-corr(3)** |
| **AV** | **L** | 37.74 | 6.E-09 | 0.33 | 7.14 | < 0.001 | 0.06 | 0.953 | -6.51 | < 0.001 | 1.42 | 0.498 | 0.01 | 1.17 | 0.756 | 1.06 | 0.756 | -0.34 | 0.756 |
|  | **R** | 19.43 | 1.E-05 | 0.29 | 3.95 | 0.001 | 6.40 | < 0.001 | 2.55 | 0.016 | 52.26 | < 0.001 | 0.29 | 5.76 | < 0.001 | 7.51 | < 0.001 | 0.76 | 0.453 |
| **CL** | **L** | 807.60 | <1.E-12 | 0.92 | 29.64 | < 0.001 | 19.96 | < 0.001 | -8.95 | < 0.001 | 381.19 | < 0.001 | 0.86 | 18.81 | < 0.001 | 16.39 | < 0.001 | -2.12 | 0.042 |
|  | **R** | 383.88 | <1.E-12 | 0.85 | 19.95 | <0.001 | 13.67 | <0.001 | -5.38 | <0.001 | 331.44 | < 0.001 | 0.84 | 17.63 | < 0.001 | 17.62 | < 0.001 | -2.12 | 0.042 |
| **CM** | **L** | 70.26 | 2.E-12 | 0.45 | 3.24 | 0.003 | -5.21 | < 0.001 | -9.91 | < 0.001 | 7.30 | 0.004 | 0.09 | -0.72 | 0.478 | -3.18 | 0.009 | -2.50 | 0.034 |
|  | **R** | 42.41 | 6.E-09 | 0.28 | -5.04 | < 0.001 | -5.39 | < 0.001 | 0.20 | 0.840 | 13.59 | < 0.001 | 0.09 | -0.26 | 0.793 | -2.91 | 0.019 | -2.64 | 0.025 |
| **LGN** | **L** | 487.55 | <1.E-12 | 0.88 | 24.72 | < 0.001 | 22.64 | < 0.001 | -3.81 | 0.001 | 205.09 | < 0.001 | 0.77 | 12.68 | < 0.001 | 16.34 | < 0.001 | 6.24 | < 0.001 |
|  | **R** | 120.22 | <1.E-12 | 0.66 | 12.69 | < 0.001 | 12.60 | < 0.001 | 4.43 | < 0.001 | 210.22 | < 0.001 | 0.73 | 13.44 | < 0.001 | 17.83 | < 0.001 | 2.23 | 0.032 |
| **MD-Pf** | **L** | 201.23 | <1.E-12 | 0.72 | -5.02 | < 0.001 | -17.10 | < 0.001 | -9.56 | < 0.001 | 196.18 | < 0.001 | 0.72 | -7.34 | < 0.001 | -18.51 | < 0.001 | -8.78 | < 0.001 |
|  | **R** | 98.05 | <1.E-12 | 0.37 | -6.34 | < 0.001 | -6.40 | < 0.001 | -2.10 | 0.044 | 62.50 | < 0.001 | 0.40 | 1.49 | 0.144 | -7.67 | < 0.001 | -6.80 | < 0.001 |
| **MGN** | **L** | 92.57 | <1.E-12 | 0.68 | 2.33 | 0.026 | -19.29 | < 0.001 | -11.61 | < 0.001 | 69.58 | < 0.001 | 0.52 | -10.09 | < 0.001 | -12.17 | < 0.001 | 0.84 | 0.406 |
|  | **R** | 89.89 | 1.E-12 | 0.62 | -9.40 | < 0.001 | -15.91 | < 0.001 | -1.12 | 0.273 | 136.97 | < 0.001 | 0.61 | -6.36 | < 0.001 | -12.71 | < 0.001 | -5.68 | < 0.001 |
| **Pul** | **L** | 414.13 | <1.E-12 | 0.80 | 21.02 | < 0.001 | 10.14 | < 0.001 | -11.14 | < 0.001 | 268.52 | < 0.001 | 0.69 | 15.26 | < 0.001 | 9.64 | < 0.001 | -4.45 | < 0.001 |
|  | **R** | 71.54 | <1.E-12 | 0.32 | 6.60 | < 0.001 | 3.22 | 0.003 | -3.80 | 0.001 | 47.19 | < 0.001 | 0.20 | 4.71 | < 0.001 | 2.67 | 0.023 | -2.46 | 0.023 |
| **VA** | **L** | 21.71 | 6.E-06 | 0.27 | -0.54 | 6.E-01 | -7.59 | 4.E-08 | -4.42 | 2.E-04 | 3.87 | 0.129 | 0.06 | -2.12 | 0.082 | -2.88 | 0.020 | -0.28 | 0.780 |
|  | **R** | 13.39 | 3.E-05 | 0.10 | 3.83 | 1.E-03 | 4.85 | 9.E-05 | -0.17 | 9.E-01 | 65.14 | < 0.001 | 0.38 | 6.41 | < 0.001 | 7.72 | < 0.001 | 2.50 | 0.017 |
| **Vla** | **L** | 195.55 | <1.E-12 | 0.72 | 19.53 | < 0.001 | 7.86 | < 0.001 | -8.23 | < 0.001 | 33.44 | < 0.001 | 0.37 | 11.15 | < 0.001 | 5.09 | < 0.001 | -3.13 | 0.004 |
|  | **R** | 8.93 | 2.E-03 | 0.13 | 3.08 | 0.009 | 3.40 | 0.005 | 1.51 | 0.140 | 1.19 | 0.498 | 0.01 | -1.08 | 0.864 | -1.03 | 0.864 | -0.19 | 0.864 |
| **VLp** | **L** | 301.78 | <1.E-12 | 0.79 | 22.87 | < 0.001 | 11.63 | < 0.001 | -5.94 | < 0.001 | 132.44 | < 0.001 | 0.65 | 16.22 | < 0.001 | 13.74 | < 0.001 | -1.76 | 0.087 |
|  | **R** | 58.68 | 8.E-10 | 0.51 | 11.13 | < 0.001 | 9.03 | < 0.001 | 4.25 | < 0.001 | 103.80 | < 0.001 | 0.63 | 16.08 | < 0.001 | 13.17 | < 0.001 | -1.26 | 0.215 |
| **VPL** | **L** | 272.42 | <1.E-12 | 0.79 | 17.76 | < 0.001 | 5.50 | < 0.001 | -14.70 | < 0.001 | 111.12 | < 0.001 | 0.56 | 7.38 | < 0.001 | 11.29 | < 0.001 | 4.08 | < 0.001 |
|  | **R** | 16.93 | 2.E-05 | 0.15 | 3.49 | 0.004 | 3.14 | 0.007 | 0.51 | 0.614 | 219.98 | < 0.001 | 0.70 | 12.66 | < 0.001 | 14.94 | < 0.001 | 3.27 | 0.002 |

Comparison of the three segmentation methods on Dice coefficients (native space) for the HCP35 and MAP35 datasets, for nuclei segmented by all three methods, relative to Morel (Krauth et al., 2010); **key:** purple: FS-T1 and FS-DTI are joint-best; orange: HIPS-THOMAS and FS-DTI are joint-best; yellow: HIPS-THOMAS and FS-T1 are joint-best; grey: no best or joint-best method; blue: FS-DTI is best; green: HIPS-THOMAS is best; AV: anteroventral nucleus; VA: ventral anterior nucleus; VLa: Ventrolateral anterior nucleus; VLp: Ventrolateral posterior nucleus; MD-Pf: mediodorsal-parafascicular nuclei; Pul: pulvinar nucleus; VPL: Ventral Posterolateral nucleus; CL: Centrolateral nucleus; CM: Centromedian nucleus; LGN: Lateral Geniculate Nucleus; MGN: Medial Geniculate Nucleus; L/R: Left, Right hemisphere; FS-T1: T_1_-based FreeSurfer segmentation (Iglesias et al., 2018); FS-DTI: FreeSurfer’s joint segmentation of thalamic nuclei from T1 scan and DTI (Tregidgo et al., 2023); HIPS-THOMAS: Thalamus Optimized Multi-atlas Segmentation using Histogram-based Polynomial Synthesis (Vidal et al., 2024); MAP35, HCP35: datasets with T1-weighted MRIs and diffusion MRI available; p-corr: the p-value was adjusted to correct for the number of multiple comparisons made (n=22), using the Holm-Bonferroni sequential correction method (Holm, 1979); ges: generalized eta squared.

## Supplementary Table 5

| **Seg.** | **Hem.** | **HCP35** | | | | | | | | | **MAP35** | | | | | | | | |
| --- | --- | --- | --- | --- | --- | --- | --- | --- | --- | --- | --- | --- | --- | --- | --- | --- | --- | --- | --- |
|  |  | **F** | **p-corr(16)** | **ges** | **HIPS-THOMAS vs. FS-T1** | | **HIPS-THOMAS vs. FS-DTI** | | **FS-T1 vs. FS-DTI** | | **F** | **p-corr(16)** | **ges** | **HIPS-THOMAS vs. FS-T1** | | **HIPS-THOMAS vs. FS-DTI** | | **FS-T1 vs. FS-DTI** | |
|  |  |  |  |  | **t(32)** | **p-corr(3)** | **t(32)** | **p-corr(3)** | **t(32)** | **p-corr(3)** |  |  |  | **t(34)** | **p-corr(3)** | **t(34)** | **p-corr(3)** | **t(34)** | **p-corr(3)** |
| **AV** | **L** | 167.71 | <1E-12 | 0.69 | 12.42 | 3.E-13 | 9.94 | 5.E-11 | -5.26 | 9.E-06 | 130.71 | <1E-12 | 0.45 | 9.25 | 2.E-10 | 16.62 | 2.E-17 | 6.05 | 7.E-07 |
|  | **R** | 74.47 | <1E-12 | 0.43 | 6.77 | 2.E-07 | 7.18 | 1.E-07 | -0.22 | 8.E-01 | 122.90 | <1E-12 | 0.37 | 10.89 | 3.E-12 | 18.75 | 5.E-19 | 0.12 | 9.E-01 |
| **CL** | **L** | 700.18 | <1E-12 | 0.93 | 28.68 | 6.E-24 | 20.43 | 1.E-19 | -8.04 | 4.E-09 | 415.00 | <1E-12 | 0.87 | 20.29 | 3.E-20 | 21.11 | 1.E-20 | -1.46 | 2.E-01 |
|  | **R** | 350.49 | <1E-12 | 0.85 | 20.51 | 2.E-19 | 12.63 | 1.E-13 | -6.57 | 2.E-07 | 333.76 | <1E-12 | 0.85 | 20.15 | 5.E-20 | 17.69 | 2.E-18 | -3.85 | 5.E-04 |
| **CM** | **L** | 245.25 | <1E-12 | 0.73 | 13.62 | 2.E-14 | 11.20 | 3.E-12 | -5.44 | 5.E-06 | 488.29 | <1E-12 | 0.82 | 23.96 | 1.E-22 | 26.18 | 1.E-23 | 2.51 | 2.E-02 |
|  | **R** | 26.72 | 8.E-06 | 0.30 | 3.44 | 2.E-03 | 6.01 | 3.E-06 | 4.39 | 2.E-04 | 214.82 | <1E-12 | 0.68 | 15.14 | 3.E-16 | 15.04 | 3.E-16 | 0.67 | 5.E-01 |
| **LGN** | **L** | 350.50 | <1E-12 | 0.86 | 21.62 | 3.E-20 | 20.52 | 1.E-19 | -1.98 | 6.E-02 | 445.58 | <1E-12 | 0.83 | 19.25 | 1.E-19 | 26.40 | 9.E-24 | 9.17 | 1.E-10 |
|  | **R** | 504.18 | <1E-12 | 0.84 | 20.75 | 1.E-19 | 17.53 | 1.E-17 | 2.92 | 6.E-03 | 575.07 | <1E-12 | 0.87 | 31.61 | 3.E-26 | 28.59 | 5.E-25 | 3.55 | 1.E-03 |
| **MD** | **L** | 156.72 | <1E-12 | 0.66 | 10.07 | 4.E-11 | -1.26 | 2.E-01 | -13.24 | 5.E-14 | 68.48 | <1E-12 | 0.45 | 10.53 | 9.E-12 | 3.84 | 5.E-04 | -10.03 | 2.E-11 |
|  | **R** | 39.54 | 1.E-09 | 0.27 | 5.10 | 3.E-05 | -0.63 | 5.E-01 | -6.47 | 9.E-07 | 118.67 | <1E-12 | 0.54 | 15.23 | 3.E-16 | 5.69 | 2.E-06 | -9.20 | 2.E-10 |
| **MGN** | **L** | 167.26 | <1E-12 | 0.77 | 12.24 | 3.E-13 | 24.93 | 5.E-22 | 2.94 | 6.E-03 | 83.65 | <1E-12 | 0.59 | 7.59 | 2.E-08 | 16.54 | 2.E-17 | 3.26 | 3.E-03 |
|  | **R** | 128.25 | <1E-12 | 0.68 | 12.78 | 8.E-14 | 17.83 | 1.E-17 | 0.50 | 6.E-01 | 109.17 | <1E-12 | 0.51 | 6.73 | 1.E-07 | 15.60 | 1.E-16 | 8.63 | 9.E-10 |
| **Pul** | **L** | 383.86 | <1E-12 | 0.79 | 20.90 | 1.E-19 | 11.95 | 5.E-13 | -9.75 | 4.E-11 | 327.41 | <1E-12 | 0.72 | 24.04 | 2.E-22 | 19.34 | 1.E-19 | -4.26 | 2.E-04 |
|  | **R** | 292.04 | <1E-12 | 0.69 | 15.25 | 9.E-16 | 9.39 | 2.E-10 | -4.18 | 2.E-04 | 193.55 | <1E-12 | 0.64 | 15.47 | 1.E-16 | 17.23 | 7.E-18 | -3.92 | 4.E-04 |
| **LT** | **L** | 231.78 | <1E-12 | 0.75 | 14.93 | 2.E-15 | 1.38 | 2.E-01 | -11.61 | 1.E-12 | 55.39 | 2.E-11 | 0.43 | 12.93 | 3.E-14 | 9.50 | 8.E-11 | 0.14 | 9.E-01 |
|  | **R** | 11.17 | 1.E-03 | 0.10 | -0.65 | 5.E-01 | 2.15 | 8.E-02 | 3.05 | 1.E-02 | 74.73 | <1E-12 | 0.40 | 13.64 | 7.E-15 | 10.83 | 3.E-12 | 1.73 | 9.E-02 |

Comparison of the three segmentation methods on Dice coefficients (native space) for the HCP35 and MAP35 datasets, for nuclei segmented by all three methods, relative to Marseille; **key:** orange: HIPS-THOMAS and FS-DTI are joint-best; grey: no best or joint-best method; green: HIPS-THOMAS is best; AV: “anteroventral” nucleus; LT: lateral thalamic nuclei; MD: “mediodorsal” nucleus; Pul: pulvinar nucleus; CL: Centrolateral nucleus; CM: Centromedian nucleus; LGN: Lateral Geniculate Nucleus; MGN: Medial Geniculate Nucleus; L/R: Left, Right hemisphere; FS-T1: T_1_-based FreeSurfer segmentation (Iglesias et al., 2018); FS-DTI: FreeSurfer’s joint segmentation of thalamic nuclei from T1 scan and DTI (Tregidgo et al., 2023); HIPS-THOMAS: Thalamus Optimized Multi-atlas Segmentation using Histogram-based Polynomial Synthesis (Vidal et al., 2024); MAP35, HCP35: datasets with T1-weighted MRIs and diffusion MRI available; p-corr: the p-value was adjusted to correct for the number of multiple comparisons made (n=16), using the Holm-Bonferroni sequential correction method (Holm, 1979); ges: generalized eta squared; Marseille: the custom-made MNI version of the atlas of deep grey matter nuclei, part of the Marseille dataset (Brun et al., 2022).

## Supplementary Table 6

| **Seg.** | **Hem.** | HCP35 | | | | | | | | | MAP35 | | | | | | | | |
| --- | --- | --- | --- | --- | --- | --- | --- | --- | --- | --- | --- | --- | --- | --- | --- | --- | --- | --- | --- |
|  |  | ANOVA | | | HIPS-THOMAS vs. FS-T1 | | HIPS-THOMAS vs. FS-DTI | | FS-T1 vs. FS-DTI | | ANOVA | | | HIPS-THOMAS vs. FS-T1 | | HIPS-THOMAS vs. FS-DTI | | FS-T1 vs. FS-DTI | |
|  |  | **F** | **ges** | **p-corr(22)** | **t(32)** | **p-corr(3)** | **t(32)** | **p-corr(3)** | **t(32)** | **p-corr(3)** | **F** | **ges** | **p-corr(22)** | **t(34)** | **p-corr(3)** | **t(34)** | **p-corr(3)** | **t(34)** | **p-corr(3)** |
| AV | L | 5.92 | 0.10 | 1.E-02 | -3.41 | 0.005 | -2.69 | 0.023 | 0.14 | 0.888 | 11.35 | 0.08 | <0.001 | 0.17 | 0.868 | -2.92 | 0.019 | -2.85 | 0.019 |
|  | R | 25.33 | 0.34 | 6.E-08 | -2.65 | 0.012 | -8.72 | <0.001 | -4.15 | <0.001 | 47.76 | 0.22 | <0.001 | -4.26 | <0.001 | -5.83 | <0.001 | -1.80 | 0.082 |
| CL | L | 377.33 | 0.86 | <1.E-12 | -17.94 | <0.001 | -43.17 | <0.001 | 0.93 | 0.359 | 38.28 | 0.43 | <0.001 | -4.08 | <0.001 | -7.07 | <0.001 | -16.46 | <0.001 |
|  | R | 323.80 | 0.83 | <1.E-12 | -16.89 | <0.001 | -28.61 | <0.001 | 0.89 | 0.383 | 45.58 | 0.47 | <0.001 | -4.65 | <0.001 | -7.78 | <0.001 | -10.65 | <0.001 |
| CM | L | 51.05 | 0.38 | 4.E-09 | -1.91 | 0.065 | 5.87 | <0.001 | 7.87 | <0.001 | 15.73 | 0.18 | <0.001 | 2.31 | 0.054 | 5.36 | <0.001 | 2.07 | 0.054 |
|  | R | 41.99 | 0.33 | 3.E-08 | 5.61 | <0.001 | 6.84 | <0.001 | 0.71 | 0.481 | 15.15 | 0.11 | <0.001 | 1.49 | 0.146 | 3.88 | 0.001 | 2.00 | 0.107 |
| LGN | L | 448.46 | 0.88 | <1.E-12 | -25.56 | <0.001 | -23.09 | <0.001 | 8.61 | <0.001 | 252.82 | 0.82 | <0.001 | -21.89 | <0.001 | -20.43 | <0.001 | -2.85 | 0.007 |
|  | R | 80.63 | 0.57 | <1.E-12 | -14.82 | <0.001 | -10.65 | <0.001 | -0.28 | 0.779 | 167.99 | 0.72 | <0.001 | -17.17 | <0.001 | -15.96 | <0.001 | -0.39 | 0.697 |
| MD-Pf | L | 272.35 | 0.78 | <1.E-12 | 9.40 | <0.001 | 20.36 | <0.001 | 7.95 | <0.001 | 199.22 | 0.79 | <0.001 | 9.97 | <0.001 | 20.26 | <0.001 | 9.30 | <0.001 |
|  | R | 116.32 | 0.46 | <1.E-12 | 5.42 | <0.001 | 8.59 | <0.001 | 4.19 | <0.001 | 66.56 | 0.46 | <0.001 | 2.01 | 0.053 | 10.61 | <0.001 | 6.05 | <0.001 |
| MGN | L | 43.29 | 0.47 | 6.E-08 | -3.12 | 0.004 | 9.19 | <0.001 | 8.26 | <0.001 | 80.13 | 0.51 | <0.001 | 9.80 | <0.001 | 6.92 | <0.001 | -3.45 | 0.002 |
|  | R | 88.42 | 0.61 | <1.E-12 | 10.73 | <0.001 | 9.55 | <0.001 | -2.56 | 0.015 | 100.22 | 0.55 | <0.001 | 6.66 | <0.001 | 9.99 | <0.001 | 2.51 | 0.017 |
| Pul | L | 382.62 | 0.82 | <1.E-12 | -19.92 | <0.001 | -7.02 | <0.001 | 13.65 | <0.001 | 193.55 | 0.57 | <0.001 | -11.74 | <0.001 | -9.31 | <0.001 | 2.56 | 0.015 |
|  | R | 71.18 | 0.21 | <1.E-12 | -6.17 | <0.001 | -3.47 | 0.003 | 1.79 | 0.083 | 105.91 | 0.33 | <0.001 | -6.53 | <0.001 | -5.85 | <0.001 | 1.29 | 0.204 |
| VA | L | 20.58 | 0.21 | 2.E-05 | -0.56 | 6.E-01 | 8.31 | 5.E-09 | 4.72 | 9.E-05 | 5.45 | 0.07 | 0.019 | 3.75 | 0.002 | 0.43 | 0.667 | -1.78 | 0.168 |
|  | R | 7.10 | 0.06 | 6.E-03 | -2.42 | 4.E-02 | -3.16 | 1.E-02 | -1.68 | 1.E-01 | 45.35 | 0.26 | <0.001 | -3.81 | 0.001 | -5.65 | <0.001 | -2.71 | 0.010 |
| Vla | L | 182.95 | 0.70 | <1.E-12 | -15.26 | <0.001 | -6.67 | <0.001 | 8.78 | <0.001 | 63.14 | 0.53 | <0.001 | -12.58 | <0.001 | -1.93 | 0.063 | 8.80 | <0.001 |
|  | R | 11.64 | 0.18 | 1.E-03 | -5.29 | <0.001 | -3.50 | 0.003 | -0.04 | 0.970 | 19.91 | 0.15 | <0.001 | -4.15 | 0.001 | 0.39 | 0.702 | 3.49 | 0.003 |
| VLp | L | 139.33 | 0.68 | <1.E-12 | -13.32 | <0.001 | -13.58 | <0.001 | 3.18 | 0.003 | 74.64 | 0.57 | <0.001 | -10.87 | <0.001 | -11.98 | <0.001 | -1.65 | 0.107 |
|  | R | 70.68 | 0.58 | 9.E-10 | -4.87 | <0.001 | -9.40 | <0.001 | -7.65 | <0.001 | 126.12 | 0.60 | <0.001 | -11.45 | <0.001 | -14.26 | <0.001 | -3.44 | 0.002 |
| VPL | L | 311.17 | 0.81 | <1.E-12 | -16.78 | <0.001 | -3.50 | 0.001 | 15.22 | <0.001 | 115.08 | 0.66 | <0.001 | -13.56 | <0.001 | -12.04 | <0.001 | -3.00 | 0.005 |
|  | R | 17.68 | 0.18 | 2.E-05 | -5.36 | <0.001 | -2.68 | 0.023 | 1.12 | 0.270 | 163.60 | 0.69 | <0.001 | -14.43 | <0.001 | -13.99 | <0.001 | -3.05 | 0.004 |

Comparison of the three segmentation methods on AHDs (native space) for the HCP35 and MAP35 datasets for nuclei segmented by all three methods, relative to Morel (Krauth et al., 2010). **key:** purple: FS-T1 and FS-DTI are joint-best; orange: HIPS-THOMAS and FS-DTI are joint-best; yellow: HIPS-THOMAS and FS-T1 are joint-best; grey: no best or joint-best method; blue: FS-DTI is best; green: HIPS-THOMAS is best; red: FS-T1 is best; AHD: average Hausdorff distance; AV: anteroventral nucleus; VA: ventral anterior nucleus; VLa: Ventrolateral anterior nucleus; VLp: Ventrolateral posterior nucleus; MD-Pf: mediodorsal-parafascicular nuclei; Pul: pulvinar nucleus; VPL: Ventral Posterolateral nucleus; CL: Centrolateral nucleus; CM: Centromedian nucleus; LGN: Lateral Geniculate Nucleus; MGN: Medial Geniculate Nucleus; L/R: Left, Right hemisphere; FS-T1: T_1_-based FreeSurfer segmentation (Iglesias et al., 2018); FS-DTI: FreeSurfer’s joint segmentation of thalamic nuclei from T1 scan and DTI (Tregidgo et al., 2023); HIPS-THOMAS: Thalamus Optimized Multi-atlas Segmentation using Histogram-based Polynomial Synthesis (Vidal et al., 2024); MAP35, HCP35: datasets with T1-weighted MRIs and diffusion MRI available. p-corr: the p-value was adjusted to correct for the number of multiple comparisons made (n=22), using the Holm-Bonferroni sequential correction method (Holm, 1979); ges: generalized eta squared.

## Supplementary Table 7

| **Seg.** | **Hem.** | **HCP35** | | | | | | | | | **MAP35** | | | | | | | | |
| --- | --- | --- | --- | --- | --- | --- | --- | --- | --- | --- | --- | --- | --- | --- | --- | --- | --- | --- | --- |
|  |  |  | **p-corr(16)** | **ges** | **HIPS-THOMAS vs. FS-T1** | | **HIPS-THOMAS vs. FS-DTI** | | **FS-T1 vs. FS-DTI** | |  | **p-corr(16)** | **ges** | **HIPS-THOMAS vs. FS-T1** | | **HIPS-THOMAS vs. FS-DTI** | | **FS-T1 vs. FS-DTI** | |
|  |  | **F** |  |  | **t(32)** | **p-corr(3)** | **t(32)** | **p-corr(3)** | **t(32)** | **p-corr(3)** | **F** |  |  | **t(34)** | **p-corr(3)** | **t(34)** | **p-corr(3)** | **t(34)** | **p-corr(3)** |
| **AV** | **L** | 135.45 | <1E-12 | 0.44 | -7.49 | 5.E-08 | -2.94 | 6.E-03 | 5.80 | 4.E-06 | 73.73 | <1E-12 | 0.33 | -8.25 | 3.E-09 | -10.82 | 4.E-12 | -5.12 | 1.E-05 |
|  | **R** | 72.47 | <1E-12 | 0.32 | -5.93 | 4.E-06 | -1.96 | 6.E-02 | 4.52 | 2.E-04 | 70.34 | <1E-12 | 0.25 | -10.94 | 2.E-12 | -11.13 | 2.E-12 | -1.32 | 2.E-01 |
| **CL** | **L** | 535.71 | <1E-12 | 0.89 | -20.73 | 8.E-20 | -53.23 | 2.E-32 | 0.44 | 7.E-01 | 52.08 | 2.E-08 | 0.50 | -5.16 | 1.E-05 | -8.48 | 1.E-09 | -16.57 | 2.E-17 |
|  | **R** | 429.63 | <1E-12 | 0.85 | -17.40 | 3.E-17 | -29.13 | 1.E-23 | 0.01 | 1.E+00 | 50.89 | 2.E-08 | 0.49 | -5.70 | 2.E-06 | -8.13 | 4.E-09 | -12.99 | 3.E-14 |
| **CM** | **L** | 220.05 | <1E-12 | 0.79 | -17.07 | 4.E-17 | -14.91 | 1.E-15 | 6.83 | 1.E-07 | 312.87 | <1E-12 | 0.84 | -18.98 | 2.E-19 | -25.43 | 3.E-23 | 1.74 | 9.E-02 |
|  | **R** | 45.95 | 7.E-10 | 0.47 | -8.00 | 8.E-09 | -8.76 | 2.E-09 | -0.50 | 6.E-01 | 258.83 | <1E-12 | 0.74 | -15.86 | 6.E-17 | -20.07 | 6.E-20 | 1.56 | 1.E-01 |
| **LGN** | **L** | 531.30 | <1E-12 | 0.89 | -27.07 | 3.E-23 | -27.82 | 2.E-23 | 4.96 | 2.E-05 | 697.12 | <1E-12 | 0.92 | -26.01 | 1.E-23 | -43.12 | 9.E-31 | -4.86 | 3.E-05 |
|  | **R** | 442.56 | <1E-12 | 0.85 | -24.98 | 4.E-22 | -21.97 | 1.E-20 | -0.19 | 9.E-01 | 578.81 | <1E-12 | 0.90 | -30.65 | 5.E-26 | -31.16 | 4.E-26 | -1.51 | 1.E-01 |
| **MD** | **L** | 217.76 | <1E-12 | 0.79 | -16.99 | 4.E-17 | -4.99 | 2.E-05 | 14.87 | 1.E-15 | 200.62 | <1E-12 | 0.78 | -17.96 | 1.E-18 | -6.02 | 8.E-07 | 19.15 | 3.E-19 |
|  | **R** | 121.70 | <1E-12 | 0.63 | -11.01 | 6.E-12 | -2.94 | 6.E-03 | 9.91 | 6.E-11 | 199.92 | <1E-12 | 0.75 | -17.02 | 1.E-17 | -6.30 | 4.E-07 | 15.46 | 1.E-16 |
| **MGN** | **L** | 411.16 | <1E-12 | 0.88 | -15.02 | 1.E-15 | -37.73 | 1.E-27 | -9.91 | 3.E-11 | 234.07 | <1E-12 | 0.81 | -9.11 | 1.E-10 | -20.86 | 2.E-20 | -12.90 | 2.E-14 |
|  | **R** | 255.25 | <1E-12 | 0.77 | -9.37 | 2.E-10 | -24.91 | 5.E-22 | -6.90 | 8.E-08 | 242.88 | <1E-12 | 0.77 | -10.82 | 1.E-12 | -18.95 | 4.E-19 | -12.95 | 2.E-14 |
| **Pul** | **L** | 190.42 | <1E-12 | 0.66 | -14.31 | 6.E-15 | -7.40 | 2.E-08 | 7.89 | 1.E-08 | 206.49 | <1E-12 | 0.51 | -16.58 | 2.E-17 | -15.09 | 2.E-16 | 2.58 | 1.E-02 |
|  | **R** | 174.65 | <1E-12 | 0.48 | -11.12 | 5.E-12 | -6.68 | 3.E-07 | 2.73 | 1.E-02 | 245.59 | <1E-12 | 0.56 | -18.40 | 9.E-19 | -16.20 | 3.E-17 | 5.45 | 4.E-06 |
| **LT** | **L** | 326.50 | <1E-12 | 0.79 | -16.25 | 2.E-16 | -3.25 | 3.E-03 | 13.10 | 4.E-14 | 164.39 | <1E-12 | 0.70 | -20.44 | 3.E-20 | -12.47 | 6.E-14 | 4.38 | 1.E-04 |
|  | **R** | 15.96 | 7.E-05 | 0.13 | -5.34 | 2.E-05 | -2.75 | 2.E-02 | 0.21 | 8.E-01 | 141.11 | <1E-12 | 0.55 | -19.72 | 1.E-19 | -10.09 | 2.E-11 | 4.44 | 9.E-05 |

Comparison of the three segmentation methods on AHDs (native space) for the HCP35 and MAP35 datasets, for nuclei segmented by all three methods, relative to Marseille; **key:** orange: HIPS-THOMAS and FS-DTI are joint-best; grey: no best or joint-best method; green: HIPS-THOMAS is best; AHD: average Hausdorff distance; AV: “anteroventral” nucleus; LT: lateral thalamic nuclei; MD: “mediodorsal” nucleus; Pul: pulvinar nucleus; CL: Centrolateral nucleus; CM: Centromedian nucleus; LGN: Lateral Geniculate Nucleus; MGN: Medial Geniculate Nucleus; L/R: Left, Right hemisphere; FS-T1: T_1_-based FreeSurfer segmentation (Iglesias et al., 2018); FS-DTI: FreeSurfer’s joint segmentation of thalamic nuclei from T1 scan and DTI (Tregidgo et al., 2023); HIPS-THOMAS: Thalamus Optimized Multi-atlas Segmentation using Histogram-based Polynomial Synthesis (Vidal et al., 2024); MAP35, HCP35: datasets with T1-weighted MRIs and diffusion MRI available; p-corr: the p-value was adjusted to correct for the number of multiple comparisons made (n=16), using the Holm-Bonferroni sequential correction method (Holm, 1979); ges: generalized eta squared; Marseille: the custom-made MNI version of the atlas of deep grey matter nuclei, part of the Marseille dataset (Brun et al., 2022).

## Supplementary Table 8

| **Method** | **Ground Truth** | **Native space** | | | | **MNI space** | | | |
| --- | --- | --- | --- | --- | --- | --- | --- | --- | --- |
|  |  | **Dice≥0.6** | | **AHD≤1.0** | | **Dice≥0.6** | | **AHD≤1.0** | |
|  |  | **HCP35** | **MAP35** | **HCP35** | **MAP35** | **HCP35** | **MAP35** | **HCP35** | **MAP35** |
| **FS-DTI** | **Morel (/22)** | 5 | 3 | 8 | 5 | 8 | 7 | 9 | 7 |
|  | **Marseille (/16)** | 5 | 6 | 6 | 5 | 6 | 6 | 6 | 4 |
| **FS-T1** | **Morel (/22)** | 2 | 2 | 8 | 8 | 7 | 6 | 8 | 10 |
|  | **Marseille (/16)** | 4 | 4 | 4 | 6 | 4 | 6 | 3 | 3 |
| **HIPS-THOMAS** | **Morel (/22)** | 7 | 8 | 11 | 13 | 10 | 13 | 15 | 15 |
|  | **Marseille (/16)** | 8 | 7 | 11 | 12 | 10 | 10 | 12 | 11 |

Number of segmentations per space (native/MNI), segmentation method (FS-T1/FS-DTI/HIPS-THOMAS), dataset (HCP35/MAP35) that have a Dice coefficient of ≥0.6 (at least “substantial” agreement/overlap) and/or a AHD≤1 (sufficiently small distance) relative to the corresponding Morel/Marseille reference/ground-truth segmentation; **key:** FS-T1: T_1_-based FreeSurfer segmentation (Iglesias et al., 2018); FS-DTI: FreeSurfer’s joint segmentation of thalamic nuclei from T1 scan and DTI (Tregidgo et al., 2023); HIPS-THOMAS: Thalamus Optimized Multi-atlas Segmentation using Histogram-based Polynomial Synthesis (Vidal et al., 2024); MAP35, HCP35: datasets with T1-weighted MRIs and diffusion MRI available; AHD: average Hausdorff distance; Morel: Krauth-Morel atlas (Krauth et al., 2010); Marseille: the custom-made MNI version of the atlas of deep grey matter nuclei, part of the Marseille dataset (Brun et al., 2022).

## Supplementary Figure 1


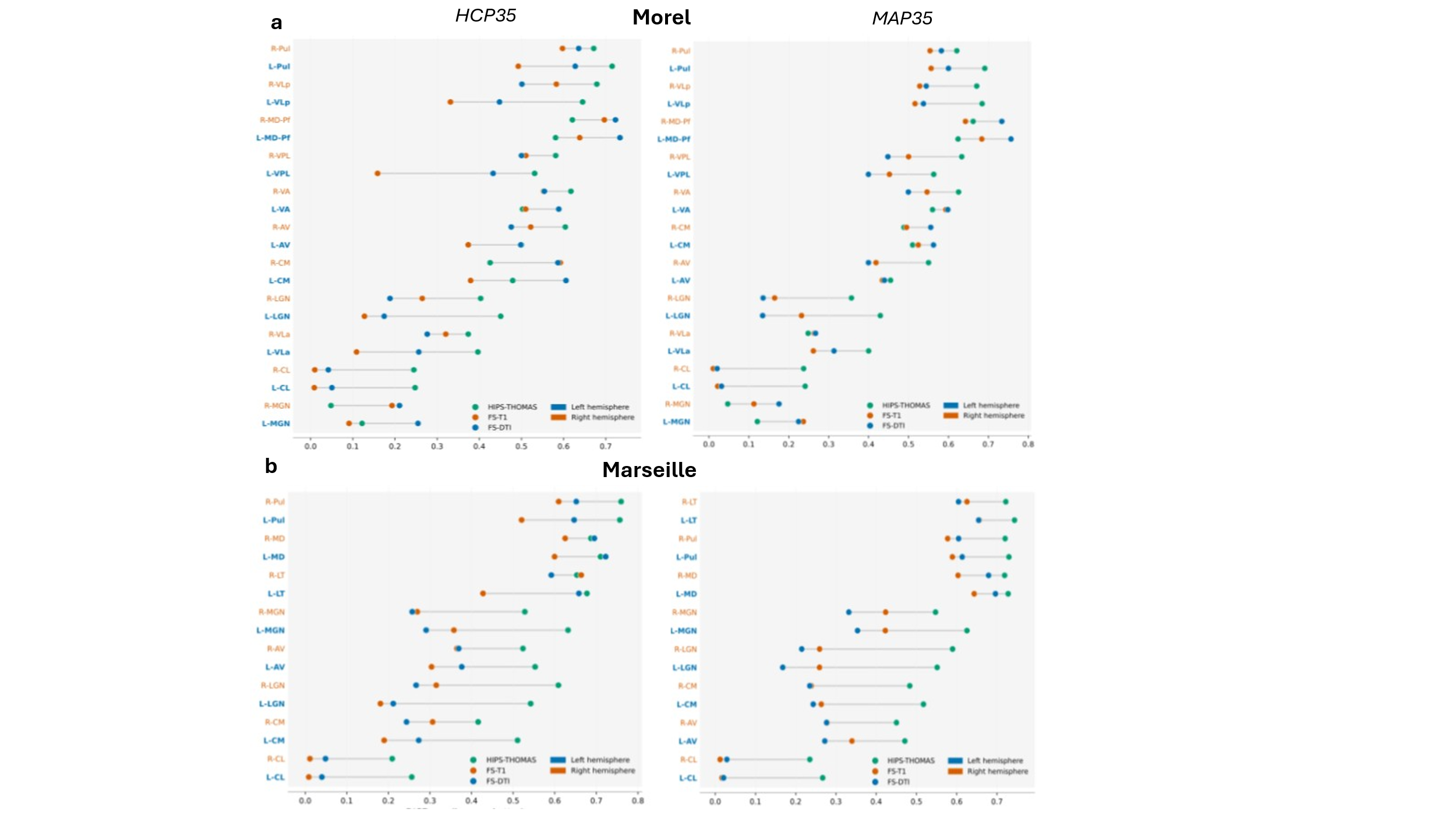
 Dumbbell plots of Dice coefficients for nuclei segmented with FS-T1, FS-DTI, and HIPS-THOMAS relative to a) Morel and b) Marseille reference segmentations (native space; see violin plots in Supplementary Figure 1); **key:** AV: anteroventral nucleus; LT: lateral nuclei (Marseille); VA: ventral anterior nucleus; VLa: Ventrolateral anterior nucleus; VLp: Ventrolateral posterior nucleus; MD-Pf: mediodorsal-parafascicular nuclei; Pul: pulvinar nucleus; VPL: Ventral Posterolateral nucleus; CL: Centrolateral nucleus; CM: Centromedian nucleus; LGN: Lateral Geniculate Nucleus; MGN: Medial Geniculate Nucleus; L/R: Left, Right hemisphere; FS-T1: T_1_-based FreeSurfer segmentation (Iglesias et al. 2018); FS-DTI: FreeSurfer’s joint segmentation of thalamic nuclei from T1 scan and DTI (Tregidgo et al. 2023); HIPS-THOMAS: Thalamus Optimized Multi-atlas Segmentation using Histogram-based Polynomial Synthesis (Vidal et al. 2024); MAP35, HCP35: datasets with T1w-MRIs and diffusion MRI available; black diamond: mean; black horizontal line: median; Morel: Krauth-Morel atlas (Krauth et al. 2010); Marseille: the custom-made MNI version of the atlas of deep grey matter nuclei, part of the Marseille dataset (Brun et al. 2022).

## Supplementary Figure 2


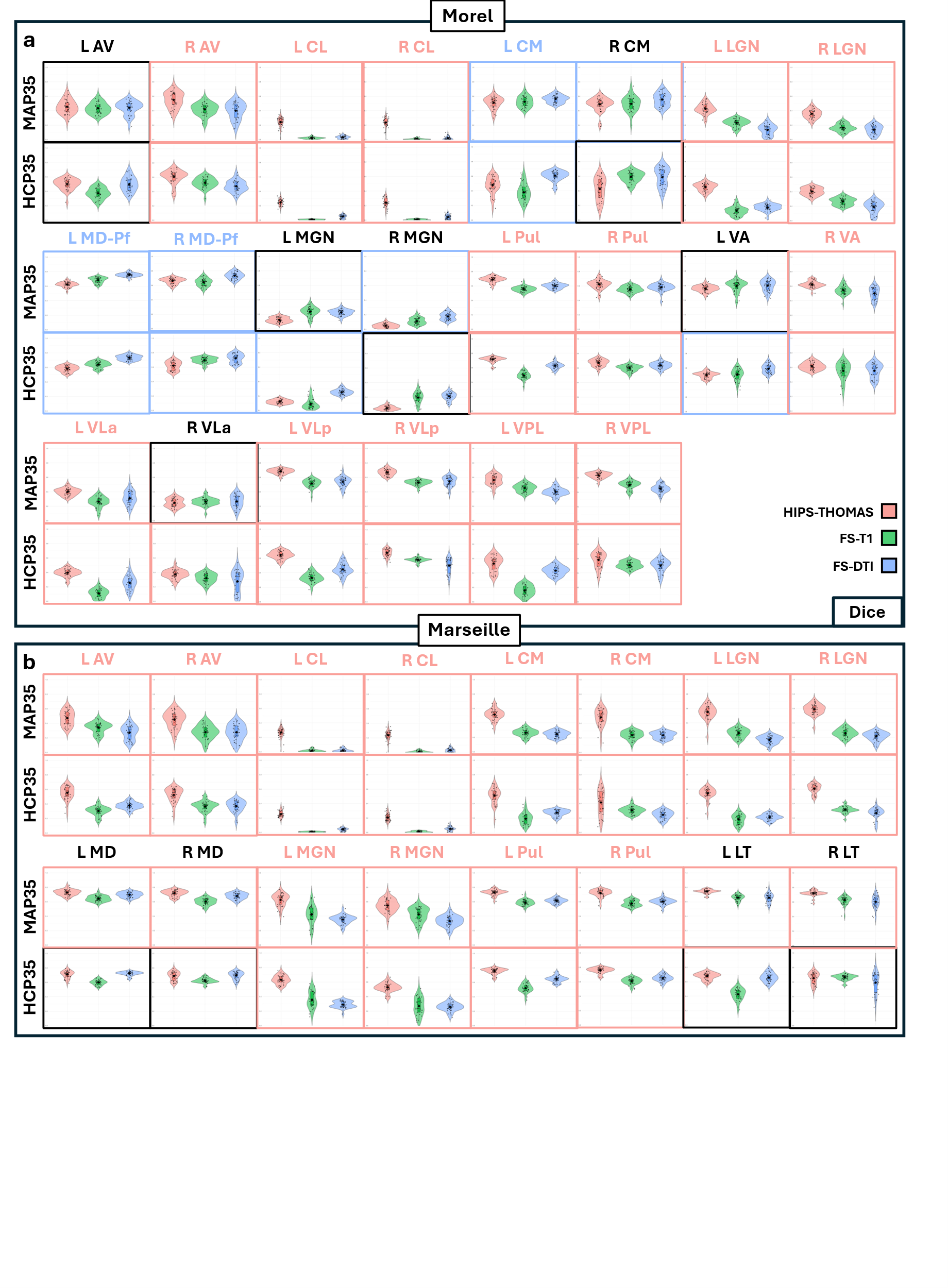
Violin plots of Dice overlap between nuclei segmented with FS-T1, FS-DTI, and HIPS-THOMAS relative to a) Morel and b) Marseille ground-truth reference segmentations (native space). Comparisons among methods that disclose significantly higher Dice coefficients for one method relative to the other two are indicated by correspondingly coloured panels; y-axis:0-1; **key:** AV: anteroventral nucleus; LT: lateral nuclei (Marseille); VA: ventral anterior nucleus; VLa: Ventrolateral anterior nucleus; VLp: Ventrolateral posterior nucleus; MD-Pf: mediodorsal-parafascicular nuclei; Pul: pulvinar nucleus; VPL: Ventral Posterolateral nucleus; CL: Centrolateral nucleus; CM: Centromedian nucleus; LGN: Lateral Geniculate Nucleus; MGN: Medial Geniculate Nucleus; L/R: Left, Right hemisphere; FS-T1: T_1_-based FreeSurfer segmentation (Iglesias et al., 2018); FS-DTI: FreeSurfer’s joint segmentation of thalamic nuclei from T1 scan and DTI (Tregidgo et al., 2023); HIPS-THOMAS: Thalamus Optimized Multi-atlas Segmentation using Histogram-based Polynomial Synthesis (Vidal et al., 2024); MAP35, HCP35: datasets with T1w-MRIs and diffusion MRI available; black diamond: mean; black horizontal line: median; Morel: Krauth-Morel atlas (Krauth et al., 2010); Marseille: the custom-made MNI version of the atlas of deep grey matter nuclei, part of the Marseille dataset (Brun et al., 2022).

## Supplementary Figure 3


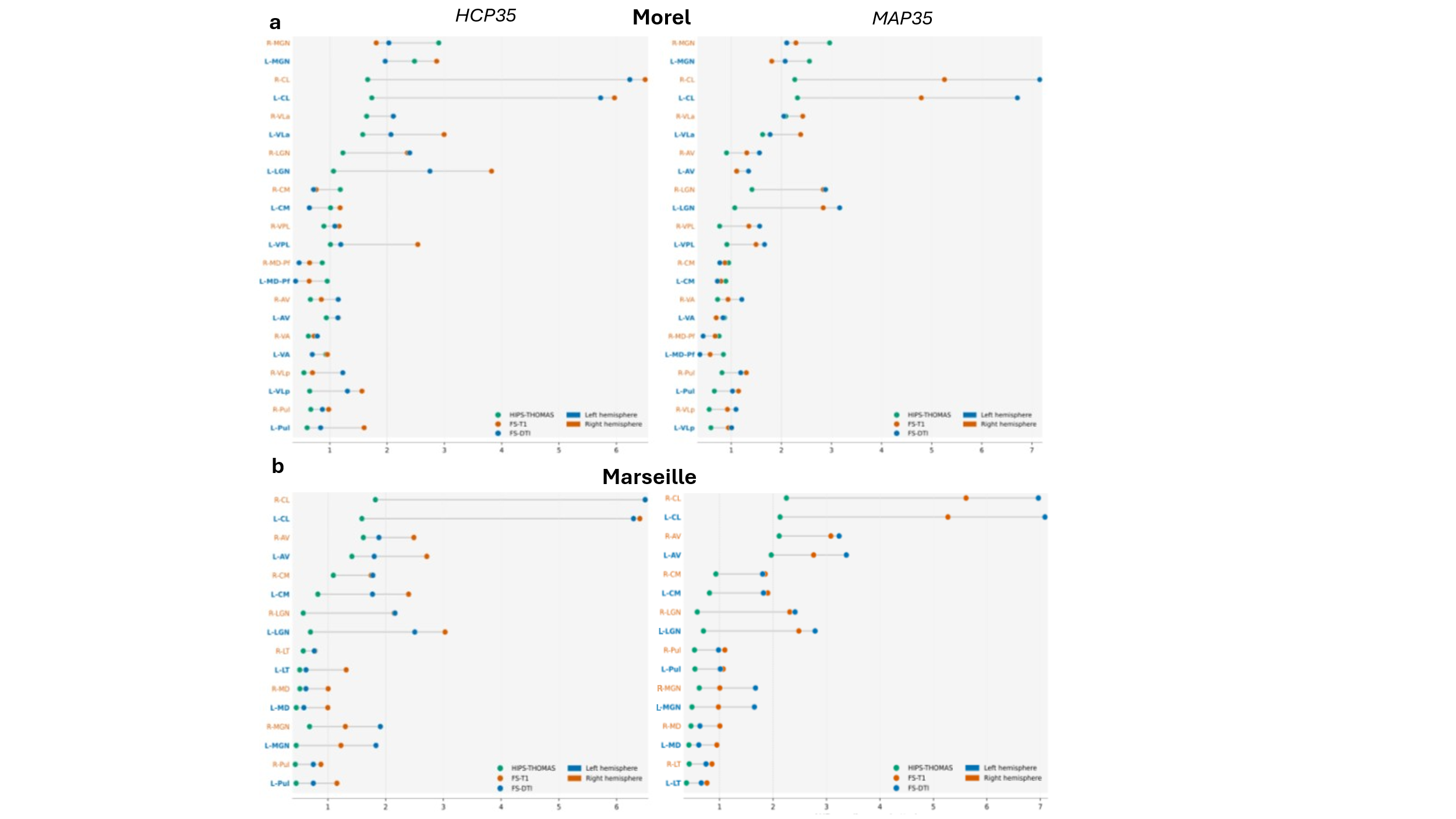


Dumbbell plots of AHD for nuclei segmented with FS-T1, FS-DTI, and HIPS-THOMAS relative to a) Morel and b) Marseille reference segmentations (native space; see violin plots in Supplementary Figure 2); **key:** AV: anteroventral nucleus; LT: lateral nuclei (Marseille); VA: ventral anterior nucleus; VLa: Ventrolateral anterior nucleus; VLp: Ventrolateral posterior nucleus; MD-Pf: mediodorsal-parafascicular nuclei; Pul: pulvinar nucleus; VPL: Ventral Posterolateral nucleus; CL: Centrolateral nucleus; CM: Centromedian nucleus; LGN: Lateral Geniculate Nucleus; MGN: Medial Geniculate Nucleus; L/R: Left, Right hemisphere; FS-T1: T_1_-based FreeSurfer segmentation (Iglesias et al. 2018); FS-DTI: FreeSurfer’s joint segmentation of thalamic nuclei from T1 scan and DTI (Tregidgo et al. 2023); HIPS-THOMAS: Thalamus Optimized Multi-atlas Segmentation using Histogram-based Polynomial Synthesis (Vidal et al. 2024); AHD: Average Hausdorff Distance; MAP35, HCP35: datasets with T1-weighted MRIs and diffusion MRI available; black diamond: mean; black horizontal line: median. Morel: Krauth-Morel atlas (Krauth et al. 2010); Marseille: the custom-made MNI version of the atlas of deep grey matter nuclei, part of the Marseille dataset (Brun et al. 2022).

## Supplementary Figure 4


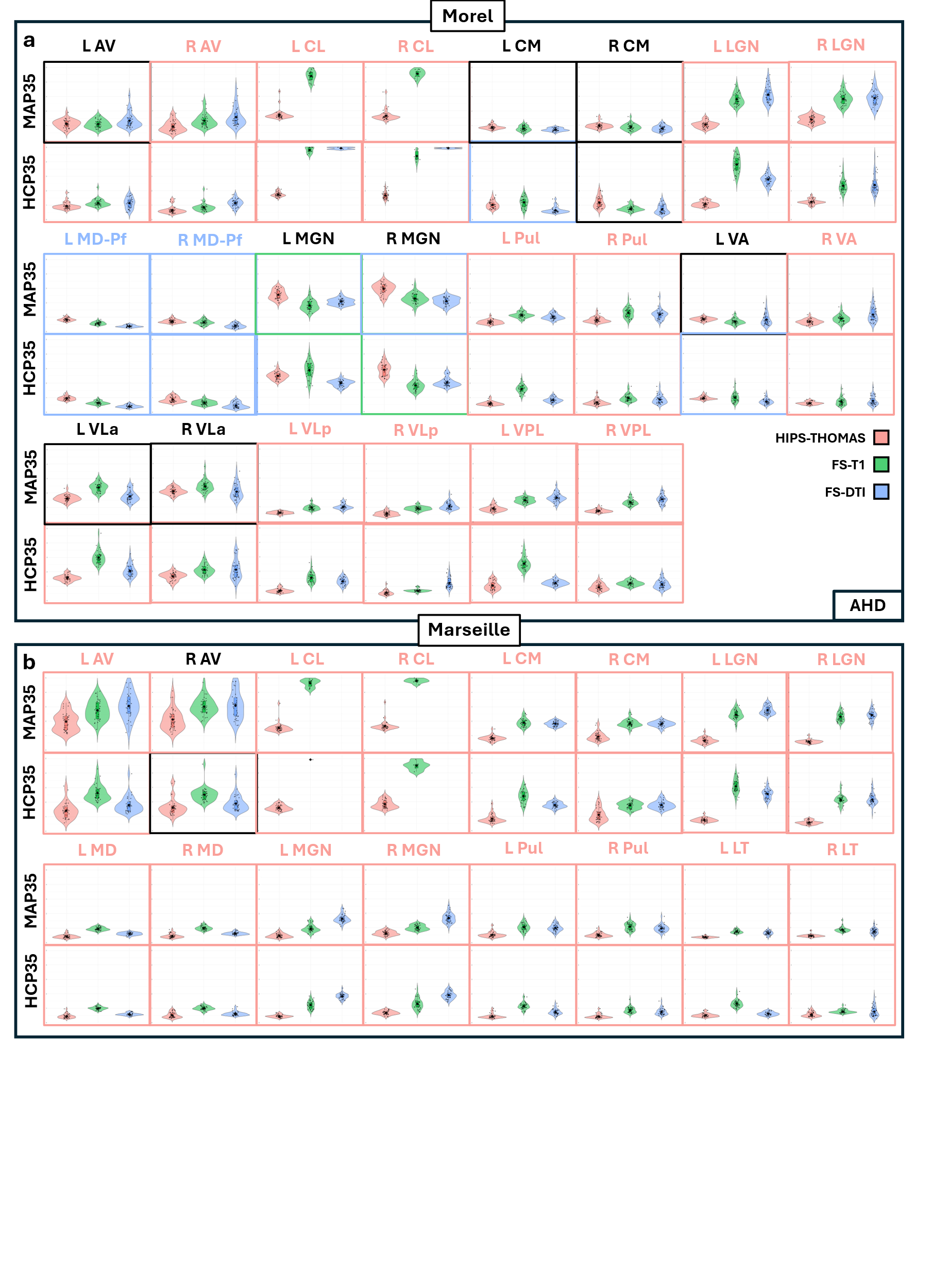


Violin plots of AHDs for native-space segmentations based on FS-T1, FS-DTI, and HIPS-THOMAS relative to a) Morel and b) Marseille ground-truth reference segmentations (y-axis:0-5mm, for display purposes). Comparisons among methods that disclose significantly lower AHD for one method relative to the other two are indicated by correspondingly coloured panels; **key:** AV: anteroventral nucleus; LT: lateral nuclei (Marseille); VA: ventral anterior nucleus; VLa: Ventrolateral anterior nucleus; VLp: Ventrolateral posterior nucleus; MD-Pf: mediodorsal-parafascicular nuclei; Pul: pulvinar nucleus; VPL: Ventral Posterolateral nucleus; CL: Centrolateral nucleus; CM: Centromedian nucleus; LGN: Lateral Geniculate Nucleus; MGN: Medial Geniculate Nucleus; L/R: Left, Right hemisphere; FS-T1: T_1_-based FreeSurfer segmentation (Iglesias et al., 2018); FS-DTI: FreeSurfer’s joint segmentation of thalamic nuclei from T1 scan and DTI (Tregidgo et al., 2023); HIPS-THOMAS: Thalamus Optimized Multi-atlas Segmentation using Histogram-based Polynomial Synthesis (Vidal et al., 2024); AHD: Average Hausdorff Distance; MAP35, HCP35: datasets with T1-weighted MRIs and diffusion MRI available; black diamond: mean; black horizontal line: median. Morel: Krauth-Morel atlas (Krauth et al., 2010); Marseille: the custom-made MNI version of the atlas of deep grey matter nuclei, part of the Marseille dataset (Brun et al., 2022).

## Supplementary Figure 5


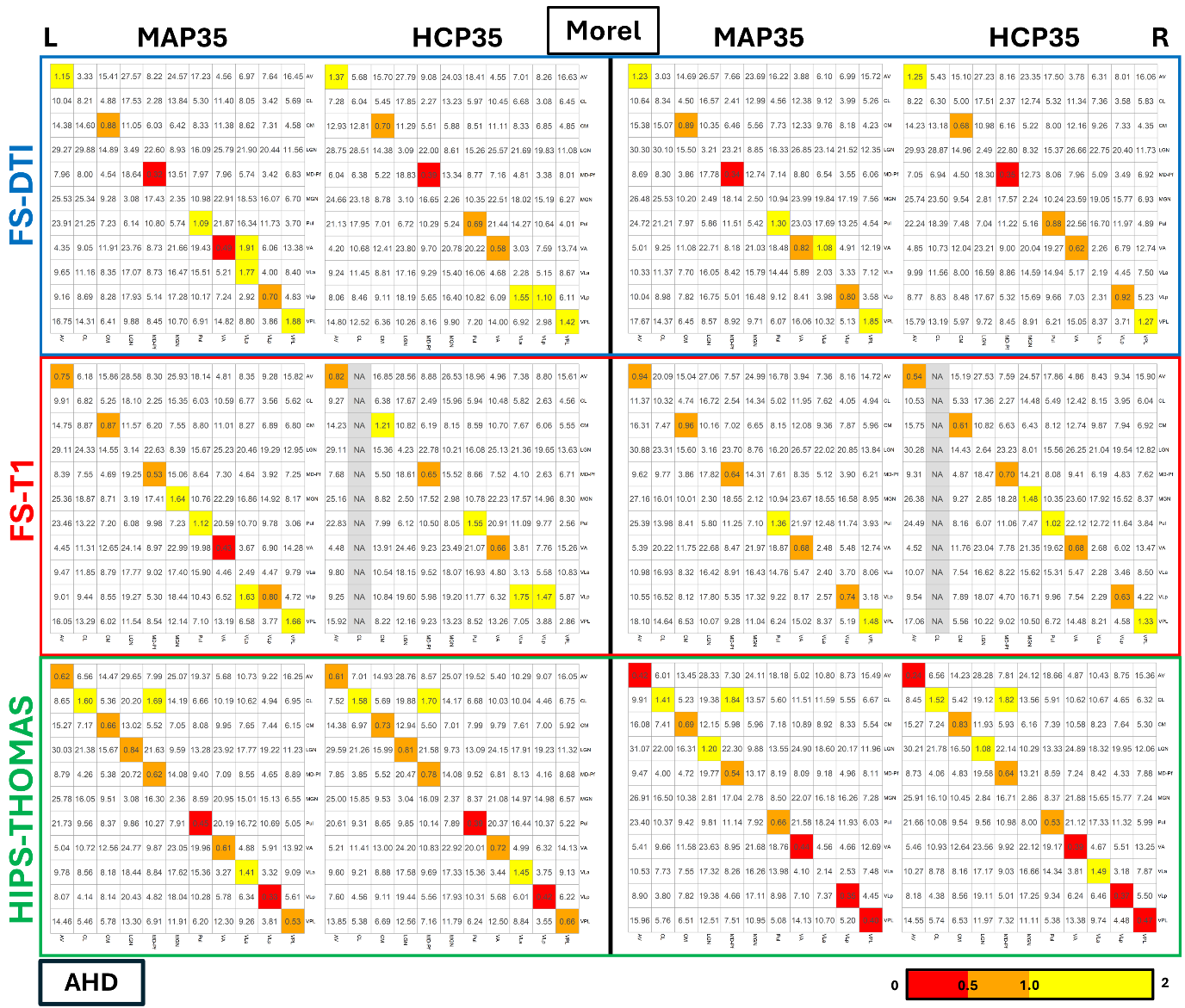


Group-level AHDs for segmentations based on FS-T1, FS-DTI, and HIPS-THOMAS from MAP35, HCP35 (MNI space) relative to Morel reference segmentations; **key:** AV: anteroventral nucleus; LT: lateral nuclei (Marseille); VA: ventral anterior nucleus; VLa: Ventrolateral anterior nucleus; VLp: Ventrolateral posterior nucleus; MD-Pf: mediodorsal-parafascicular nuclei; Pul: pulvinar nucleus; VPL: Ventral Posterolateral nucleus; CL: Centrolateral nucleus; CM: Centromedian nucleus; LGN: Lateral Geniculate Nucleus; MGN: Medial Geniculate Nucleus; L/R: Left, Right hemisphere; FS-T1: T_1_-based FreeSurfer segmentation (Iglesias et al. 2018); FS-DTI: FreeSurfer’s joint segmentation of thalamic nuclei from T1 scan and DTI (Tregidgo et al. 2023); HIPS-THOMAS: Thalamus Optimized Multi-atlas Segmentation using Histogram-based Polynomial Synthesis (Vidal et al. 2024); AHD: Average Hausdorff Distance; Morel: Krauth-Morel atlas (Krauth et al. 2010).

## Supplementary Figure 6


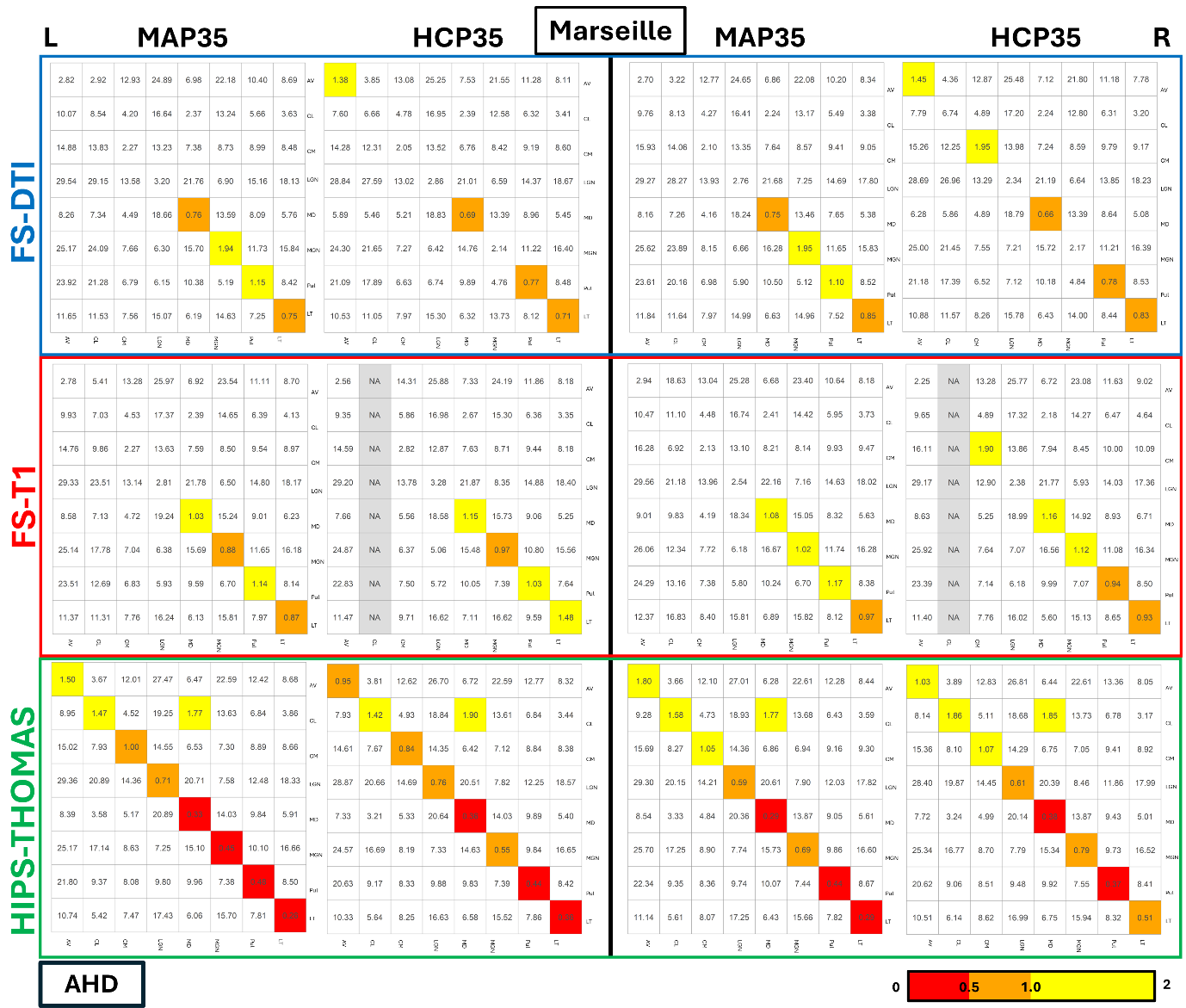


Group-level AHDs for segmentations based on FS-T1, FS-DTI, and HIPS-THOMAS from MAP35, HCP35 (MNI space) relative to Marseille reference segmentations; **key:** AV: anteroventral nucleus; LT: lateral nuclei (Marseille); VA: ventral anterior nucleus; VLa: Ventrolateral anterior nucleus; VLp: Ventrolateral posterior nucleus; MD-Pf: mediodorsal-parafascicular nuclei; Pul: pulvinar nucleus; VPL: Ventral Posterolateral nucleus; CL: Centrolateral nucleus; CM: Centromedian nucleus; LGN: Lateral Geniculate Nucleus; MGN: Medial Geniculate Nucleus; L/R: Left, Right hemisphere; FS-T1: T_1_-based FreeSurfer segmentation (Iglesias et al. 2018); FS-DTI: FreeSurfer’s joint segmentation of thalamic nuclei from T1 scan and DTI (Tregidgo et al. 2023); HIPS-THOMAS: Thalamus Optimized Multi-atlas Segmentation using Histogram-based Polynomial Synthesis (Vidal et al. 2024); AHD: Average Hausdorff Distance; Marseille: the custom-made MNI version of the atlas of deep grey matter nuclei, part of the Marseille dataset (Brun et al. 2022).

# References

Brun, G., Testud, B., Girard, O. M., Lehmann, P., de Rochefort, L., Besson, P., Massire, A., Ridley, B., Girard, N., Guye, M., Ranjeva, J.-P., & Le Troter, A. (2022). Automatic segmentation of deep grey nuclei using a high-resolution 7T magnetic resonance imaging atlas—Quantification of T1 values in healthy volunteers. *European Journal of Neuroscience*, *55*(2), 438–460. https://doi.org/10.1111/ejn.15575

Holm, S. (1979). A simple sequentially rejective multiple test procedure. *Scandinavian Journal of Statistics*, *6*(6), 65–70. https://doi.org/10.2307/4615733

Iglesias, J. E., Insausti, R., Lerma-Usabiaga, G., Bocchetta, M., Van Leemput, K., Greve, D. N., van der Kouwe, A., Fischl, B., Caballero-Gaudes, C., & Paz-Alonso, P. M. (2018). A probabilistic atlas of the human thalamic nuclei combining ex vivo MRI and histology. *NeuroImage*, *183*, 314–326. https://doi.org/10.1016/j.neuroimage.2018.08.012

Krauth, A., Blanc, R., Poveda, A., Jeanmonod, D., Morel, A., & Székely, G. (2010). A mean three-dimensional atlas of the human thalamus: Generation from multiple histological data. *NeuroImage*, *49*(3), 2053–2062. https://doi.org/10.1016/j.neuroimage.2009.10.042

Tregidgo, H. F. J., Soskic, S., Olchanyi, M. D., Althonayan, J., Billot, B., Maffei, C., Golland, P., Yendiki, A., Alexander, D. C., Bocchetta, M., Rohrer, J. D., & Iglesias, J. E. (2023). Domain-agnostic segmentation of thalamic nuclei from joint structural and diffusion MRI. In *Medical Image Computing and Computer Assisted Intervention – MICCAI 2023. MICCAI 2023. Lecture Notes in Computer Science* (Vol. 14227, pp. 247–257). Springer. https://doi.org/10.1007/978-3-031-43993-3_24

Vidal, J. P., Danet, L., Péran, P., Pariente, J., Bach Cuadra, M., Zahr, N. M., Barbeau, E. J., & Saranathan, M. (2024). Robust thalamic nuclei segmentation from T1-weighted MRI using polynomial intensity transformation. *Brain Structure & Function*, *229*, 1087–1101. https://doi.org/10.1007/s00429-024-02777-5
